# Widespread cortical functional disconnection in gliomas: an individual network mapping approach

**DOI:** 10.1093/braincomms/fcac082

**Published:** 2022-04-08

**Authors:** Erica Silvestri, Manuela Moretto, Silvia Facchini, Marco Castellaro, Mariagiulia Anglani, Elena Monai, Domenico D’Avella, Alessandro Della Puppa, Diego Cecchin, Alessandra Bertoldo, Maurizio Corbetta

**Affiliations:** 1 Department of Information Engineering, University of Padova, 35131 Padova, Italy; 2 Padova Neuroscience Center, University of Padova, 35129 Padova, Italy; 3 Department of Neuroscience, University of Padova, 35128 Padova, Italy; 4 Neuroradiology Unit, University Hospital of Padova, 35128 Padova, Italy; 5 Neurosurgery, Department of NEUROFARBA, University Hospital of Careggi, University of Florence, 50139 Florence, Italy; 6 Department of Medicine, Unit of Nuclear Medicine, University of Padova, 35128 Padova, Italy; 7 Venetian Institute of Molecular Medicine, 35129 Padova, Italy

**Keywords:** functional connectivity, resting-state networks, glioma, disconnection, single subject

## Abstract

Assessment of impaired/preserved cortical regions in brain tumours is typically performed via intraoperative direct brain stimulation of eloquent areas or task-based functional MRI. One main limitation is that they overlook distal brain regions or networks that could be functionally impaired by the tumour. This study aims (i) to investigate the impact of brain tumours on the cortical synchronization of brain networks measured with resting-state functional magnetic resonance imaging (resting-state networks) both near the lesion and remotely and (ii) to test whether potential changes in resting-state networks correlate with cognitive status. The sample included 24 glioma patients (mean age: 58.1 ± 16.4 years) with different pathological staging. We developed a new method for single subject localization of resting-state networks abnormalities. First, we derived the spatial pattern of the main resting-state networks by means of the group-guided independent component analysis. This was informed by a high-resolution resting-state networks template derived from an independent sample of healthy controls. Second, we developed a spatial similarity index to measure differences in network topography and strength between healthy controls and individual brain tumour patients. Next, we investigated the spatial relationship between altered networks and tumour location. Finally, multivariate analyses related cognitive scores across multiple cognitive domains (attention, language, memory, decision making) with patterns of multi-network abnormality. We found that brain gliomas cause broad alterations of resting-state networks topography that occurred mainly in structurally normal regions outside the tumour and oedema region. Cortical regions near the tumour often showed normal synchronization. Finally, multi-network abnormalities predicted attention deficits. Overall, we present a novel method for the functional localization of resting-state networks abnormalities in individual glioma patients. These abnormalities partially explain cognitive disabilities and shall be carefully navigated during surgery.

## Teaser

Silvestri *et al*. developed a new method to investigate resting-state brain networks in patients with gliomas. They showed that gliomas cause broad alterations of network topography occurring mainly in structurally normal regions outside the lesion. These abnormalities partially explain cognitive disabilities and shall be carefully navigated during surgery.

## Graphical Abstract

**Graphical Abstract:**
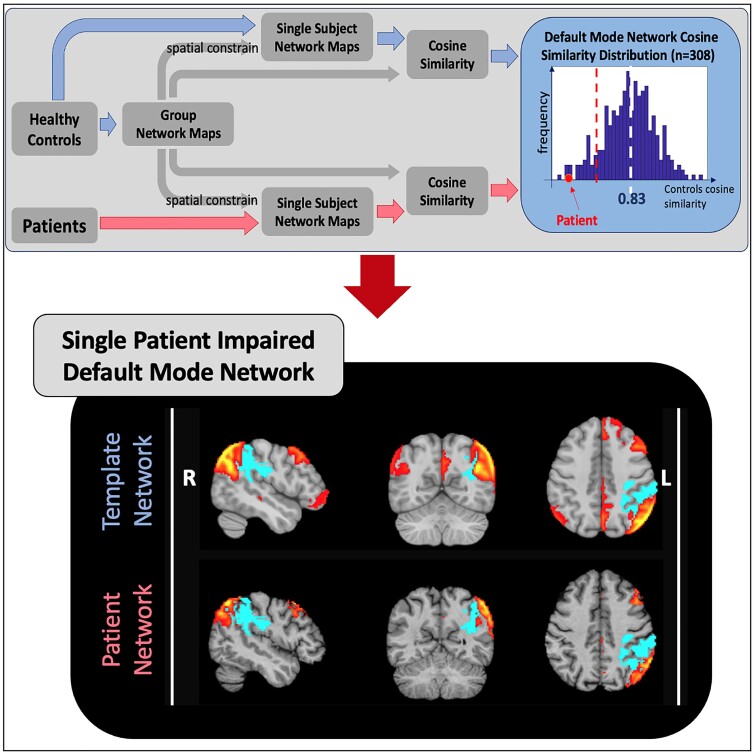

## Introduction

Primary glioma tumours in adults represent a heterogeneous group of expansive lesions of the central nervous system.^1^ Neurosurgical resection is the first-line therapeutic approach to the treatment of brain tumours.^2^ Growing evidence suggests that gross total resection, as compared with subtotal resection, leads to an improved patient overall survival and progression-free survival both in case of high- and low-grade tumours.^3–5^ Nevertheless, the benefits of a prolonged survival must be balanced against the risks of significant decrements of quality of life of both patient and caregivers due to permanent neurological dysfunctions following extensive resections. In this regard, the goal of the surgical planning is to preserve critical functional regions, or eloquent areas, and structural connections while removing most of the tumour.^2^

The intraoperative direct brain stimulation during awake surgery is the gold standard for functional mapping of eloquent areas. However, this method requires specific instrumentation and increases the risk of epileptic seizures during the surgery.^6,7^ In addition, functional MRI (fMRI) has proven to be a valid non-invasive and highly sensitive alternative tool for localizing distinct eloquent cortical and subcortical areas before surgery in glioma.^7^ Task-based fMRI is widely used^8^ as a pre-surgical mapping tool^2^ and potentially could play a role in monitoring treatment and prognosis.^7,9^ Unfortunately, task-based fMRI is demanding for the patient, it is heavily influenced by the patient’s performance, and it is not easy to implement in a standard clinical setting.^7,8,10^

Recent studies^11–14^ have proposed the use of resting-state (rs-)fMRI as a reliable technique to overcome these limitations proving its effectiveness in the mapping of eloquent areas of motor^15^ and language^2,16^ functions. Published studies have restricted the analysis to mapping of eloquent functions in the perilesional area or specific functional networks such as the default mode network (DMN).^13,17,18^ Nevertheless, distal regions or networks could be also functionally impacted by brain tumours^18,19^ through mechanisms of structural or functional disconnection. There have been only a handful of studies addressing this issue. Nenning *et al*.^20^ and Stoecklein *et al*.^21^ introduced two different indexes to quantify functional connectivity at the voxel level and have reported significant functional changes at a distance from the tumour, as well as a significant correlation between functional connection alterations and clinical variables such as patient’s cognitive status or tumour grade.

In the present study, we investigated the effect of gliomas on the brain’s main functional networks measured with rs-fMRI (resting-state networks, RSNs), both near and far from the lesion. We were interested in examining if functional abnormalities involve predominantly the region near the tumour, the oedema region or structurally normal tissue. Patients often present to the hospital in the acute phase with focal neurological deficits that improve with high-dose steroid therapy. This may indicate a role of the oedema region in modulating function. In addition, we were interested in examining if potential RSN abnormalities may be related to pre-surgery neuropsychological status.

We developed a novel whole-brain approach, based on independent component analysis (ICA), to robustly detect impaired networks at the single-patient level. This approach does not need a prior identification of specific regions of interest such as in seed-based functional connectivity^19,22^ or in recent studies developing disconnections biomarkers investigating the connectivity between the tumour area and the rest of the brain.^20,21^ The approach is data-driven and exploits the richness of the data by comparing independent components (ICs) identified in individual patients with template components defined in a separate group of healthy subjects. Single-patient abnormalities in functional connectivity are determined based on both topology and strength of correlation as compared with healthy controls (HCs). Individual maps can be used for neuro-navigation, and multi-network abnormalities can be related to cognitive status.

## Materials and methods

### Study cohort

Pre-surgical data of 24 patients (11 female, mean age 58.1 ± 16.4 years) with *de novo* brain tumours were collected at the University Hospital of Padova. All participants have regularly taken anticonvulsants for control of epilepsy and corticosteroids. The protocol had been approved by the local Ethics Committee of the University Hospital of Padova and carried out in accordance with the 1964 Helsinki declaration and its later amendments. Informed consent was obtained from all participants.

As HCs, we used a subset of the publicly available MPI-Leipzig Mind-Brain-Body data set.^23^ This data set comprises rs-fMRI scans on *n* = 318 subjects acquired on a 3 T Siemens Magnetom Verio scanner with a T_2_*-weighted gradient-echo echo planar imaging (EPI) sequence [repetition time (TR) = 1400 ms, echo time (TE) = 39.4 ms, flip angle (FA) = 69°, field of view (FOV) = 202 ×  202 mm, voxel size = 2.3 mm ×  2.3 mm × 2.3 mm, volumes = 657, multiband acceleration factor (MBAccFactor) = 4, iPAT = 0, phase encoding direction antero-posterior]. Ten HCs were discarded due to scanner artefacts or data unavailability. The final HC group consisted of 308 subjects (125 females; mean age: 36.96 ± 18.40 years).

### MRI acquisition

Data were acquired on a 3 T Siemens Biograph mMR scanner equipped with a 16-channel head–neck coil. Anatomical imaging included T_1_-weighted (T1w) 3D magnetization-prepared rapid acquisition gradient-echo (TR = 2400 ms, TE = 3.24 ms, TI = 1000 ms, FA = 8°, FOV = 256 × 256 mm, voxel size = 1 mm × 1 mm × 1 mm) images acquired both before and after contrast agent injection, a 3D T_2_-weighted image (TR = 3200 ms, TE = 535 ms, FOV = 256 × 256 mm, voxel size = 1 mm × 1 mm × 1 mm), a 3D fluid attenuation inversion recovery (TR = 5000 ms, TE = 284 ms, TI = 1800 ms, FOV = 256 × 256 mm, voxel size = 1 mm × 1 mm × 1 mm) image. In addition, functional imaging comprised rs-fMRI EPI scans (TR = 1260 ms, TE = 30 ms, FA = 68°, FOV = 204 × 204 mm, voxel size = 3 mm × 3 mm × 3 mm, volumes = 750, MBAccFactor = 2, iPAT = 0, phase encoding direction antero-posterior) and two spin echo-EPI acquisitions with reverse phase encoding (TR = 4200 ms, TE = 70 ms, FOV = 204 × 204 mm, voxel size = 3 mm × 3 mm × 3 mm, MBAccFactor = 1) for EPI distortion correction purposes.

### Neuropsychological assessment

A neuropsychological battery was administered covering different cognitive domains. It included the Oxford Cognitive Screen (OCS^24^) and the Esame Neuropsicologico Breve 2.^25^

For each subject, the raw scores were converted to *Z*-scores according to standardized normative values. Next, the tests were divided into four domains based on the measured cognitive domain: memory, language, executive functions and attention (Supplementary Table 3). For each subject, we obtained a global *Z*-score for each domain averaging the *Z*-score of single tests assigned to that domain. Positive scores indicate good performance.

Two patients failed to complete the entire battery of neurological tests and thus were disregarded in the analysis linking behaviour to RSN changes.

### Tumour segmentation

The anatomical images of each patient were linearly registered to the patient naïve T1w image with the Advanced Normalization Tools (ANTs,^26^ v. 2.0.1). Using these images, two masks were manually delineated through the ITK-SNAP software (http://www.itksnap.org/) by an expert neuroradiologist with more than 5 years of experience (M.A.). The first mask, the tumour mask (TM), included the tumour core (contrast agent enhancing and non-enhancing regions) and the necrosis. The second mask, the tumour and oedema area mask (TM + *O*), was created by adding the oedema area to the TM.

### MRI pre-processing

Imaging data of both patients and HCs underwent an analogous structural and functional pre-processing. Data were pre-processed with an in-house pipeline using the following software: ANTs, Statistical Parametric Mapping 12 (SPM12, v. 7219) and FMRIB’s Software Library (FSL, www.fmrib.ox.ac.uk/fsl, v. 6.0.3).

Structural pre-processing consisted of bias field correction (*N4BiasFieldCorrection*^27^), skull-stripping (*Multi-Atlas Skull Stripping*^28^), tissue segmentation (into grey and white matter and cortico-spinal fluid, with the *unified segmentation tool*^29^ of SPM12) and diffeomorphic non-linear registration (as implemented in ANTs *SyN* algorithm) to the symmetric MNI 2009c atlas. In the patient group, the last step was performed excluding the TM + *O*^30^ area.

Functional pre-processing of rs-fMRI data included slice timing, readout distortion (FSL’s TOPUP^31^) correction, affine realignment of volumes to the central image of the acquisition (FSL’s *mcflirt*^32^), a non-linear mapping to the symmetric MNI152 atlas exploiting the subject-specific T1w (via FSL’s boundary based registration^33^) and high pass filtering (cut-off frequency: 0.008 Hz). Since the MBfactor of the EPI acquisition sequence differed between the patients and HCs data set, spurious variance related to scanner artefacts was regressed out from the patients’ pre-processed data using an ICA-based approach.^34^ Thus, the ICs related to sequence MBfactor or broad head movement artefacts were manually identified and regressed out from the original pre-processed data.

Finally, to quantify the subject-specific head motion during the scan, we computed the frame-wise displacement as defined in Power *et al.*^35^ and compared it between the patient population and controls cohort with the Wilcoxon rank-sum test to ensure that the two groups did not significantly differ in terms of head motion.

### Assessment of RSN alterations

**Figure 1:**
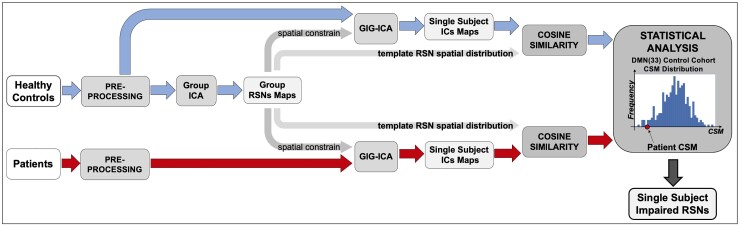
**Analysis workflow**. Pipeline followed to assess patients’ functional alterations.

RSNs were identified at the individual level by means of the ICA and compared in terms of the spatial pattern and magnitude with the control group. The workflow is depicted in Fig. 1.

Functional pre-processed data were analysed with the Group ICA of fMRI toolbox (GIFT)^36^ and custom codes written in Matlab (MATLAB 2020b, The MathWorks, Inc., Natick, MA, USA).

Since the ICA approach can pose issues of reproducibility and correspondence between individual and controls networks or single-ICs,^37,38^ we exploited the group information-guided ICA (GIG-ICA) back-reconstruction framework^38^ as implemented in the GIFT toolbox to accurately retrieve the single-subject (SS) RSNs spatial pattern.^39^ Recent work^38,39^ indicates that this approach leads to the best correspondence of ICs across subjects and higher sensitivity to group differences. In brief, this framework involves exploiting the group information captured by standard ICA at the group level as guidance to compute SS ICs using a multi-objective optimization strategy. The analysis works in two stages: first, group ICs (i.e. the *template ICs*) are obtained using standard group ICA; second, the *template ICs* are used as references (or spatial prior) in a new one-unit ICA with spatial reference using a multi-objective optimization solver (GIG-ICA). The GIG-ICA simultaneously optimizes the independence of each subject-specific IC, measured by negentropy, as well as the correspondence between each subject-specific IC and each *template IC*.^40^ This minimization ensures that each IC in a patient or in a HC is compared with the correct respective *template IC* (or normative component).

Accordingly, we first defined the high-resolution *template ICs* in a cohort of HCs. According to a study by Du *et al*.,^40^ the GIG-ICA approach is not so sensitive to the model order selection and performs well under the case of poor data quality (i.e. low signal-to-noise ratio, SNR) and quantity (less time points); however, the accuracy in detecting the ICs is positively related to the SNR (i.e. higher the SNR, better the accuracy). Therefore, to limit the impact of mixing data sets with different data quality and quantity, we identified the data set provided by Mendes *et al*.^23,41^ as control data set. It was in fact acquired with similar characteristics to our glioma data set (i.e. rs-fMRI acquisition sequence with similar voxel size, TR and duration).

Since movement introduce artefacts that significantly impact the estimation of functional connectivity in terms of both connectivity matrices^42^ and ICs,^37,43^ we applied the group-level spatial ICA to a subset of HC controls (*n* = 140) homogeneously selected in the available age ranges (*n* = 35, range 20–30 years; *n* = 27, 30–40 years; *n* = 8, 40–50 years; *n* = 7, 50–60 years; *n* = 41, 60–70 years and *n* = 22, 70–80 years) and with an average frame-wise displacement^35^ (0.14 ± 0.05 mm) comparable with that of the patients (0.16 ± 0.06 mm). The high model order decomposition^44^ was performed by using the Infomax algorithm and setting the number of ICs to 180. To confirm the stability of the result, the ICA was repeated 10 times within the ICASSO^45^ framework and a central solution was selected using the modes of the component cluster. The goodness of the final decomposition was evaluated with the cluster stability/quality (*Iq*) index returned from ICASSO and compared with literature reports.

Then, following,^46^ the group ICs were manually labelled into artefactual (i.e. related to head motion, physiological noise, MBfactor, and so on) or intrinsic connectivity network, the *components*, by visually inspecting their spatial maps and time courses power spectra.

Afterwards, components were grouped into 10 functional networks (RSNs) according to their spatial pattern: visual network (VIS), sensorimotor network (SMN), auditory network (AUD), cingulo-opercular network (CON), dorsal attention network (DAN), fronto-parietal network (FPN), DMN, cognitive control network (CCN), frontal network (FRN) and language network (LANG). Specifically, we first binarized each component by applying a threshold to the *Z*-score map of *Z* = 1 and then computed their normalized spatial overlap with the networks depicted by two functional atlases: the Yeo Atlas (seven networks),^47^ and the Gordon Atlas,^48^ and assigned the component to the best matching RSN (i.e., the network with the highest normalized spatial overlap). In addition, RSN components with poor overlap with the previous two atlases (normalized spatial overlap lower than 50%) were localized using the database NeuroSynth (https://www.neurosynth.org) using the peak activation position to find the best matching network. Finally, for the LANG, we have referred to the spatial patterns reported by both task-based^49^ and resting-state studies.^50,51^

It is important to highlight that this approach defines a high number of components that are consistent across subjects and are grouped according to known cortical parcellation schemes. This high dimensionality that isolates several subcomponents of each RSN allows for a finer match with individual components in patients that may be abnormal in terms of strength or topography.

Once defined the high-resolution *template ICs*, we used the selected group-level components as spatial constraints within the GIG-ICA back-reconstruction step to accurately retrieve their spatial pattern at the individual level in both patients and all subjects belonging to the control group (*n* = 308). At the end, for each patient and HC, each back-reconstructed component consisted of a spatial *Z*-score map reflecting the network’s coherent activity across space.

To determine whether a patient’s component was impaired, we examined its topography and magnitude as compared with the IC distribution in the HC group.

In the HCs, the spatial variability of the ICs was measured by quantifying the variability between individual and template components in each healthy subject. For each component, the similarity between the *template* and the SS IC is quantified with the cosine similarity (CS) measure, computed as

(1) $$\mathrm{CS}({\mathrm{comp}}_{i})=\frac{{\mathrm{Map}}_{\mathrm{template}}({\mathrm{comp}}_{i})\cdot {\mathrm{Map}}_{\mathrm{SS}}({\mathrm{comp}}_{i})}{\left|\left|{\mathrm{Map}}_{\mathrm{template}}({\mathrm{comp}}_{i})\right||\cdot |\left|{\mathrm{Map}}_{\mathrm{SS}}({\mathrm{comp}}_{i})\right|\right|}$$

where comp_*i*_ is the *ith* component and Map(RSN_*i*_) is the vectorization of the component’s spatial map. We choose this index because it is sensitive to both the spatial pattern and the magnitude (i.e. connectivity strength) of the component. For each component, we derived then the statistical description of the CS distribution in the HC group (Fig. 1, last panel).

To note that, as we were interested in the peak activation of each IC, both in controls and patients the CS was not computed at the whole-brain level but within a representative mask of the component.

Since we were interested in the peak activation of each IC, both in controls and patients, the CS was not computed at the whole-brain level but within a representative mask of the component. The component mask was obtained by (i) thresholding the template IC at *Z*-score = 1, a very tolerant threshold that took into consideration possible spatial shifts in the position of the IC due to distortions caused by the tumour; (ii) filtering spurious voxels, i.e. only clusters with at least 200 connected voxels were analysed. We also removed cerebellar voxels based on the Neuromorphometrics Inc. atlas (http://www.neuromorphometrics.com/), as this structure was not fully covered in all patients. Next, we vectorized the component spatial map to examine only voxels that fell both within the template and individual IC mask. To determine alterations of specific ICs in single patients, we computed the CS between the two vectors as in equation (1) and ran a permutation test to compare for each IC the CS of each patient against the distribution of CS values in HCs.

ICs marked as abnormal, that is significantly different from the HC CS distribution, were manually checked to ensure that they were not present outside of the mask used to compute the CS. Given the threshold used, we did not observe any of 166 abnormal ICs (based on the CS method) to be shifted by the tumour outside of the mask used for the CS computation, but the use of this type of mask could be a limitation in the application of the proposed method if a manual check is not performed.

It was not possible to include the cerebellum in these analyses because in most subjects it fell outside the FOV of the rs acquisition. Even subcortical areas were excluded as for some patients they showed significant resting-state signal dropout issues.

### Overlap between tumour and altered RSNs

We examined whether the tumour location or the extent of oedema affected the component topography, by computing for all patients the percentage overlap between the altered components and (i) the TM, (ii) the oedema and (iii) the remaining normal-appearing brain tissues. This computation was performed both considering separately each component and using a unique mask that included all altered components.

Since for some patients altered components may be partially or completely missing, the overlap with the tumour was calculated using the expected map of the component, i.e. that obtained through the group-level ICA. Hence, as shown in Supplementary Fig. 1, after detecting the impaired components, for each patient we went back to the *template* ICs and extracted for each altered component its expected maps (i.e. the *template* component map). Then, we binarized these maps using the same approach as described above for the component mask definitions. Finally, we created the ‘expected’ patient-altered RSNs mask as the union of the obtained binary maps.

The percentage normalization was computed using the abnormal mask extent as reference, as follows:

(2) $$Overlap_{\text{\%}}=\;\frac{{\mathrm{alteredRSN}}_{\mathrm{mask}}\;\cap^{\;}\;tissue_{mask}}{{\mathrm{alteredRSN}}_{\mathrm{mask}}}$$

### Statistical analysis

#### Altered RSNs

For each patient, each component that fell within the template IC (see previously) was labelled as significantly altered after a permutation test comparing the patient’s CS with the HCs’ CS distribution. The number of permutations was 50 000 and the significance level was set to 0.05. As 140 of the 308 controls were used to build the *template ICs* and thus a higher CS with the *template* was expected for these subjects, to minimize the impact of this group, in each permutation we compared the CS distribution of 200 out of 308 HCs against the single-patient CS value. We employed a threshold of three standard deviations as strength of evidence against the null hypothesis that the patient’s CS belonged from the HC distribution.

#### Relationship between RSNs and neuropsychological score

Finally, a multivariate analysis was conducted to test whether a statistically significant relationship existed between components changes and patients’ cognitive performance. With a multiple linear regression analysis, we separately predicted the aggregate score of each neuropsychological score (NPS) domain from the components’ delta CS (ΔCS*_σ_*), which quantified the distance of each patient’s single component from the control group topography as follows:

(3) $$\Delta CS_{\sigma }({\mathrm{RSN}}_{i})=\frac{CS_{\mathrm{SS},{\mathrm{RSN}}_{i}}-\mu (CS_{\mathrm{group},{\mathrm{RSN}}_{i}})}{\sigma (CS_{\mathrm{group},{\mathrm{RSN}}_{i}})}$$

where *μ* and *σ* were, respectively, the sample mean and standard deviation of the CS of the HC group. The final linear model took as dependent variable the single NPS aggregate score and as independent variables the ΔCS*_σ_* of each component. The model fit was performed using the linear least square with negative constraints estimator. The non-negativity constraint was imposed for two reasons: the first is that we hypothesized that the distance from the group topography of a component should contribute positively to explain NPS impairment. The second is that this approach intrinsically implements a feature selection and thus avoids model overfitting ensuring that only actually informative variables were included in the final model.

The goodness of model fit and estimates were assessed by means, respectively, of the squared Pearson’s correlation (*R*^2^) between the model prediction and the dependent variable, the evaluation of the precision of the estimates as coefficient of variation (CV). Then, we considered only those models that achieved an *R*^2^ of 0.5 and CVs of the estimates lower than 200% as representative of significant relationship.

### Data availability

The data that support the findings of this study are available from the corresponding author, upon reasonable request.

## Results

### Study cohort

Patients main demographical and clinical information are summarized in Table 1. A detailed description of the single-patient characteristics is provided in Supplementary Table 1.

**Table 1:** Patients’ demographics and clinical data

Age	58.1 ± 16.4 years
*Gender*	
Female	11
Male	13
*Tumour histology*	
Astrocytoma	1
Diffuse astrocytoma	1
Glioblastoma	15
Gliosarcoma	1
Glioneuronal neoplasm	2
Oligodendroglioma	1
Other	3
*Tumour grade*	
I	1
II	3
III	2
IV	17
n.a.	1
*IDH1 mutation status*	
Wild-type	14
Mutated	6
n.a.	4
*Tumour site*	
Left	14
Right	6
Bilateral	4

IDH, isocitrate dehydrogenase gene; n.a., not available.

**Figure 2:**
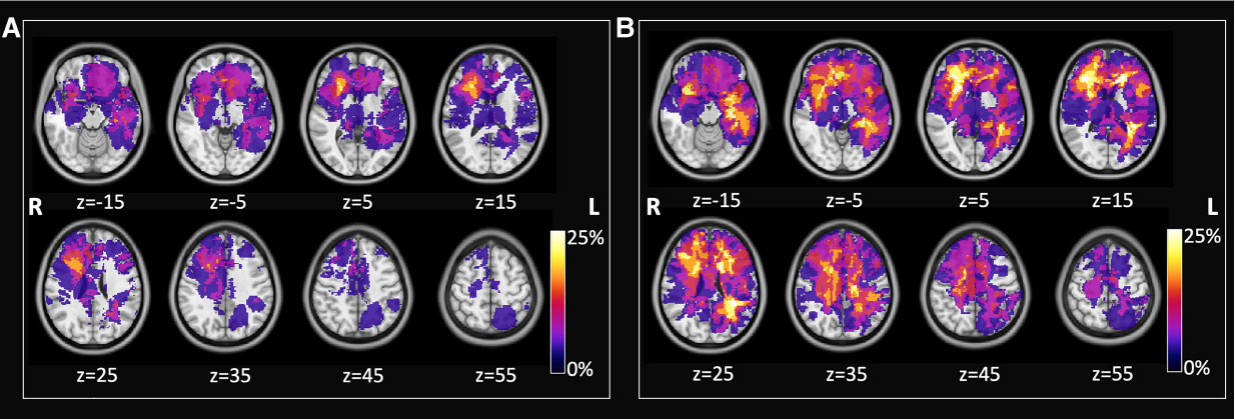
**Lesion frequency map across patients.** (**A**) Frequency map of tumour core, (**B**) map of tumour lesions including oedema area. Maps are over imposed to the MNI atlas (grey scale). Radiological convention.

Figure 2 shows the frequency maps of the lesions in the patient population. The two reported maps refer to the TM (Fig. 2A) and to the TM + *O* masks (Fig. 2B). The distribution is sparse with tumours involving predominantly the right frontal and left temporal lobes, with a low spatial overlap (maximum value 20.8% of patients for the TM and 33.3% of patients for the TM + *O*).

No statistically significant differences were found between the patients’ and HCs’ group in head motion during rs-fMRI acquisition: the median frame-wise displacement was, respectively, of 0.15 ± 0.04 and 0.14 ± 0.04; the rank-sum test *P* = 0.16.

### Neuropsychological assessment

The individual score tests were aggregated in four major cognitive domains: language, attention, memory and executive functions. Supplementary Table 3 reports normalized in *Z-scores* and the aggregate score for each domain, whereas Supplementary Fig. 4 shows the frequency distribution across patients of the aggregate scores. Memory was the most frequently impaired (9/22 patients with scores below normal), followed by executive function deficits (8/22), language (8/22) and attention (4/22).

### Assessment of RSN alterations

Overall, 45 different ICs were identified. The obtained decomposition was highly reliable and reproducible with a cluster *Iq* of the selected components that ranged between 0.736 and 0.996 (in line with findings in a study by Saha *et al*.^52^).

Each component was assigned to a specific RSN based on the overlap with the Yeo Atlas (seven networks)^47^ and the Gordon Atlas^48^ (VIS, SMN, AUD, CON, DAN, FPN and DMN) or by mean of metanalyses provided by the NeuroSynth database (CCN, FRN, LANG). Eventually, we obtained 11 *components* in the VIS, four in the SMN, one in the AUD, three in the CON, three in the DAN, four in the FPN, six in the DMN, nine in the CCN, three in the FRN and one in the LANG RSNs. The spatial pattern of the different components is depicted in Supplementary Fig. 2.

**Figure 3:**
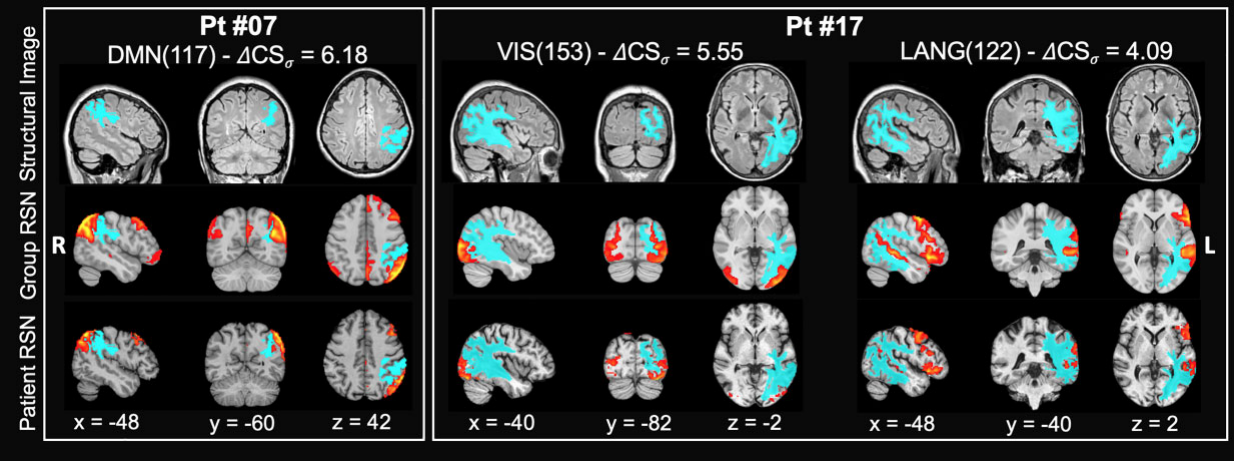
**Example of altered RSNs in two representative patients.** The patients were affected by IDH1 mutated high-grade glioblastomas in the left hemisphere. Structural image: fluid attenuation inversion recovery image with superimposed the segmentation of the tumour and oedema (light blue). Group RSN: T1w MNI atlas with RSN HCs group average component (red-yellow scale). Patient RSN: patient individual altered component. Left panel: DMN component [DMN(117)] in Patient #07. Right panel: VIS(153) and LANG(122) component in Patient #17. ΔCS*_σ_* = delta CS, i.e. distance from the group average. Radiological convention.

In our patient cohort, several components revealed significantly different topography to that observed in the HCs. Figure 3 shows examples of how the tumour affects the RSN spatial distribution in two patients suffering from IDH1 mutated high-grade glioblastoma of the left hemisphere. The first patient (#07) has a lesion in the white matter of the left inferior parietal lobule (IPL). The DMN (117) component is strongly altered with a ΔCS*_σ_* = −6.18. A relatively normal left IPL cortical component overlying the lesion contrasts with the loss of the praecuneus, contralateral right IPL and ipsilateral left lateral and mesial frontal components. This pattern is consistent with the disconnection of white matter pathways connecting the left IPL with the other nodes of the DMN. The second patient (#17) has a large lesion involving occipital, parietal and temporal white matter with a cortical involvement. The VIS(153) component in HCs is bilateral in the occipital cortex and extends dorsally along the intraparietal sulcus. In the patient, we observe some preservation of the ipsilateral occipital component but a loss of contralateral occipital and bilateral parietal components (which leads to a ΔCS*_σ_* of −5.55), again consistently with white matter disconnection. The same patient also shows a significant alteration of the LANG network (ΔCS*_σ_* = 4.09) with a weakened superior temporal and frontal components of the network.

These three examples highlight the effect of gliomas on the correlated activity of cortical regions that are distant from the site of the tumour but are functionally connected through altered white matter pathways.

**Figure 4:**
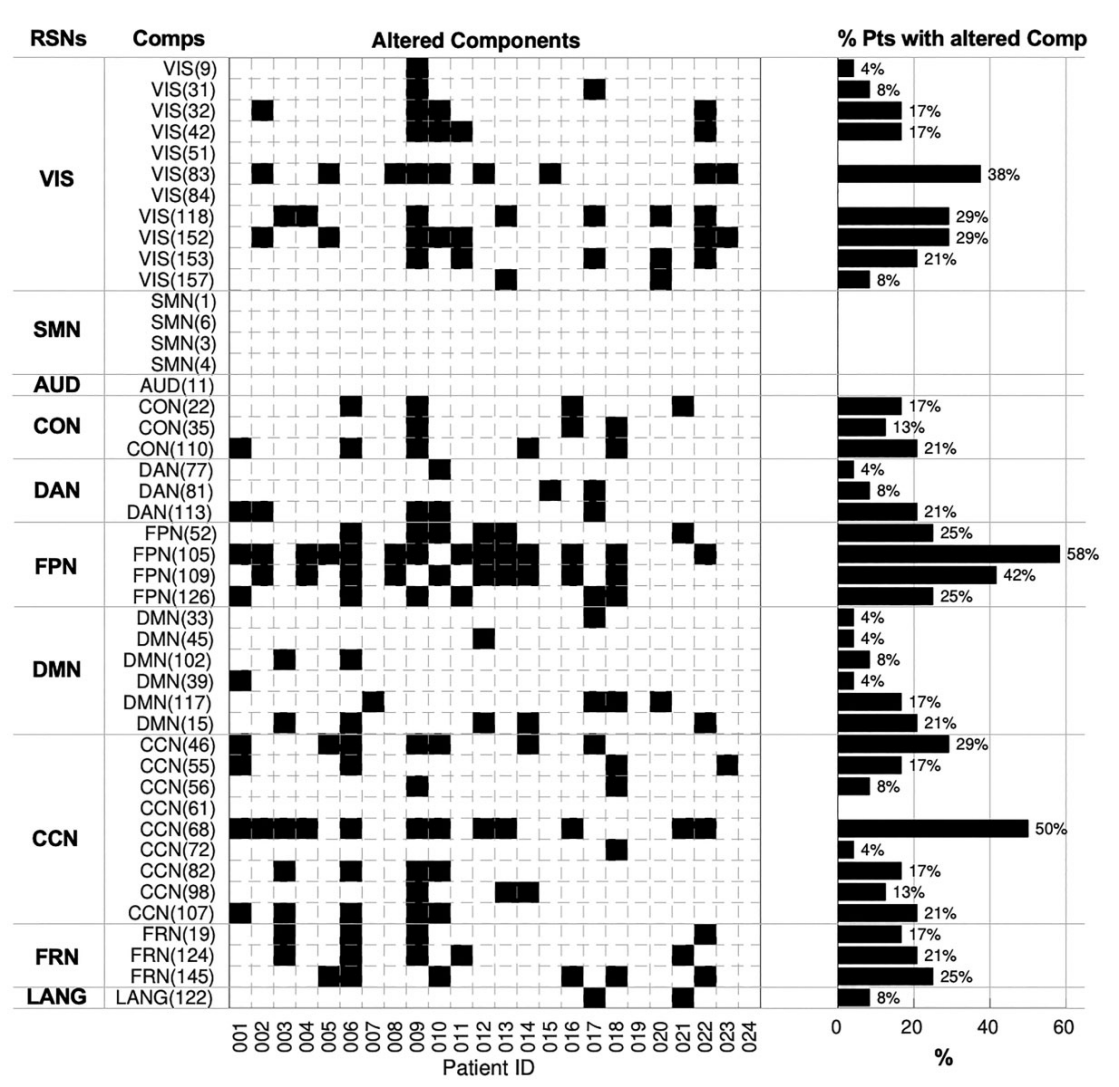
**Altered RSNs.** On the left, the matrix reports significant alterations, marked as black squares. Rows represent specific independent components (Comps) organized by networks they belong to and columns represent single patients. On the right, for each component, the bar plot shows the percentage of patients with that component damaged. VIS, visual network; SMN, sensorimotor network; AUD, auditory network; CON, cingulo-opercular network; DAN, dorsal attention network; FPN, fronto-parietal network; DMN, default mode network; CCN, cognitive control network; FRN, frontal network; LANG, language network.

For each patient, we quantified the significant changes in the network topography (Fig. 4). Overall, we detected in each patient a relatively high number of altered components ranging from 0 to 23 (mean value 6.9 ± 5.2). Aggregating these findings by RSNs and ranking them by % of patients with at least one component affected, we found that 70.8% of the patients had alterations in the FPN, 70.8% in CCN, 62.5% in VIS, 41.7% in DMN, 41.6% in FRN, 29.2% in CON, 25% in DAN and 8.3% in LANG network.

When averaging across patients, tumours caused alterations in 35.9% of FPN components, 21.7% of FRN, 17.4% of CON, 17.4% of CCN, 15.4% of VIS, 11.6% of DAN, 10.1% of DMN and 8.7% of LANG network. Interestingly, no altered components occurred in the SMN and AUD network.

Although the relatively higher frequency of tumours in the frontal lobe explains the abnormalities of RSN components in FPN, CCN, CON, networks with a strong prefrontal representation, more surprising is the high frequency of abnormal VIS component, given their location in the occipital lobe, the least frequently involved structurally and farthest away from the most common frontal and temporal locations.

It is also notable that in this group of glioma patients, enrolled for a neurosurgery, no functional abnormalities were detected in the SMN and very few in the LANG network. This might reflect a selection bias towards lesions that are ‘safer’ to operate when they do not cause motor or language impairments after surgery.

### Overlap between tumour and altered RSNs

The single component percentage overlap is shown in Supplementary Fig. 3. Overall, we found an overlap with the TM mask ranging between 0 and 22.1%, oedema between 0 and 11.0% and with normal tissue between 74.1 and 100%. Overall, the VIS, DAN and LANG network show the least overlap with the TM with a maximum overlap of 6.3, 2.4 and 4.3%, respectively.

To quantify the impact of tumour at the level of individual subject, Supplementary Table 2 reports the results of the overlap with tumour tissue (TM, oedema, normal tissue) patient by patient. The mean percentage overlap was quite small with both TM (2.6% ± 2.3%) and oedema (1.0% ± 1.5%). The range of overlap was also quite small: TM (0–8.2%) and oedema (0–4.9%). Most of the network alterations involved normally appearing tissue with a mean overlap of 96.3% ( ± 2.7%) and in the range of 91–100%.

These findings suggest that network alterations involve regions near the tumour or within the oedema in a limited way but more robustly distant functionally connected regions.

To examine this issue, we measured spatial pattern alteration of each component (quantified by the ΔCS*_σ_*) and the overlap between these components and TM. There was a weak negative statistically significant Spearman correlation between altered components ΔCS*_σ_* and the overlap with the TM mask (*ρ* = −0.18, *P* = 0.04) and no statistically significant correlation with the overlap with the oedema area (*ρ* = −0.04, *P* > 0.05). These findings indicate that RSN components were not altered in relation to the proximity with the tumour or overlap with the oedema.

### Relationship between RSNs and NPSs

**Figure 5:**
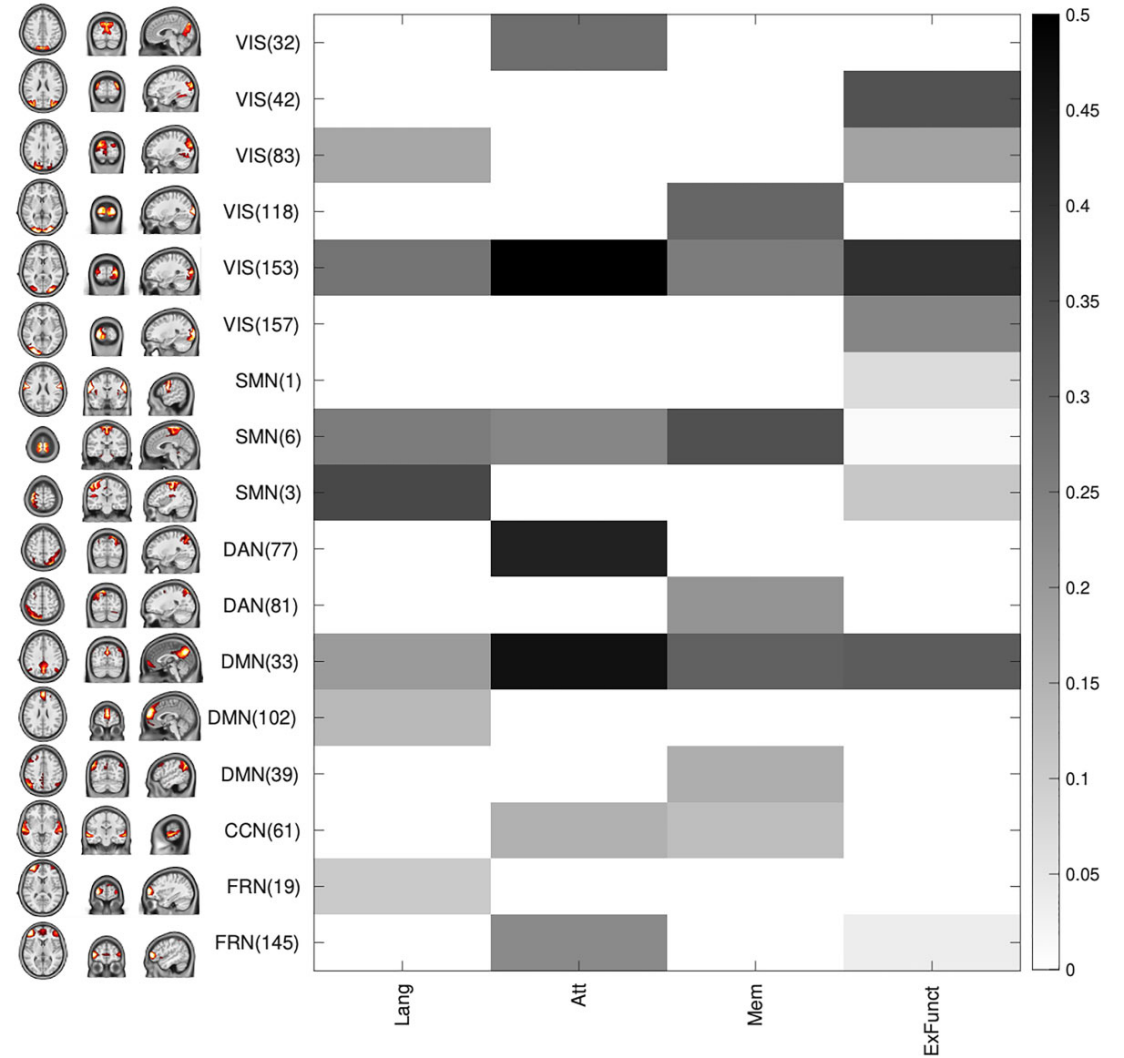
**Relationship between changes in component’s spatial pattern and neuropsychological aggregate scores.** The matrix reports the correlation between component’s delta CS −ΔCS*_σ_* (on the rows, with group spatial pattern of the component on the left) and NPS aggregate scores (on the columns) for the components selected with the multivariate analysis for at least one functional domain. In grey scale, only the correlation values obtained for predictors included in the linear model are reported (Lang, language; Att, attention; Mem, memory; ExFunc, executive function).

**Figure 6:**
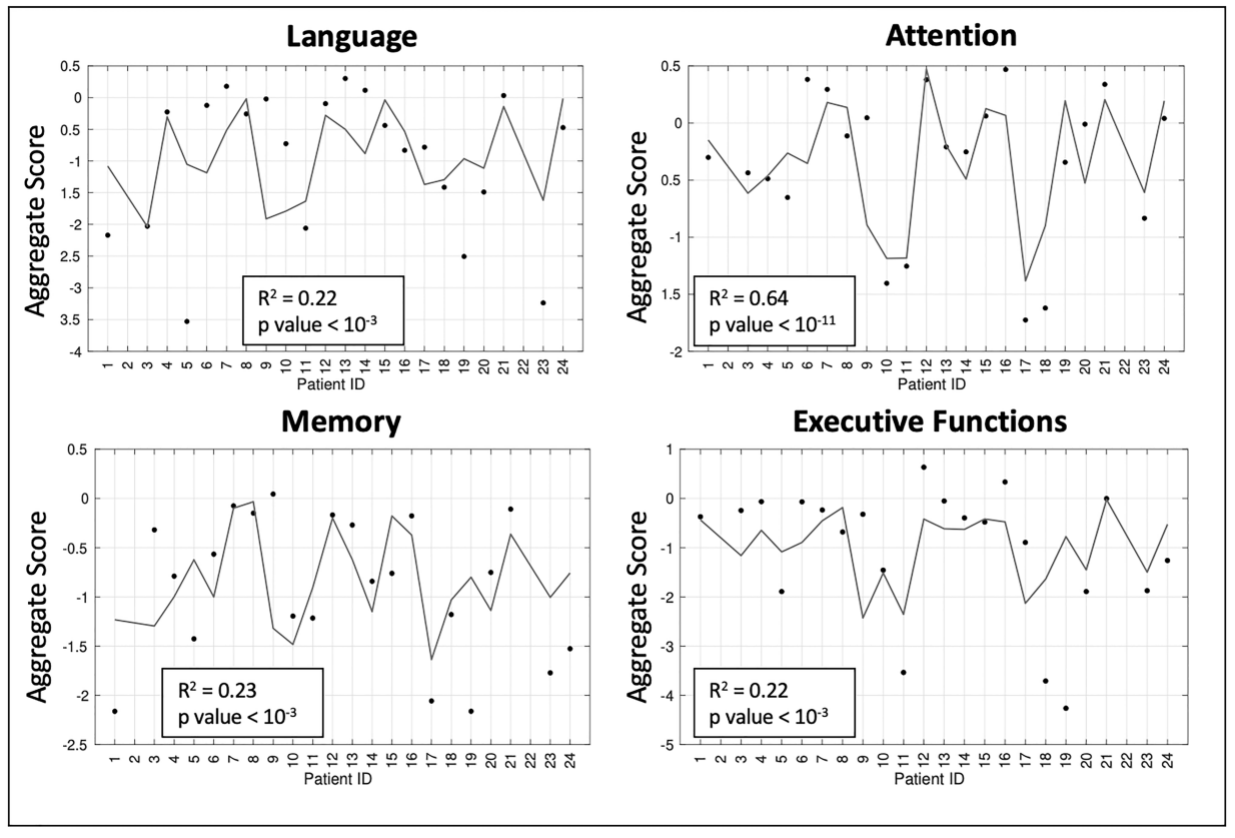
**Neuropsychological aggregate scores model predictions.** The four boxes show the measured aggregate scores (black dots) and the linear prediction of the four tested neuropsychological functional domains.

Results of multiple regression analyses run on the neuropsychological pre-surgery scores are shown in Figs 5 and 6. The non-negative least square model fitting approach leaded to a considerable model sparsification: in fact, only 7 of 45 RSN components were selected to separately predict LANG, attention and memory aggregate scores and 9 of 45 RSN components for the executive functions aggregate score. Figure 5 shows the relationship between the patient’s ΔCS*_σ_* of the selected RSNs and the NPS aggregate score. In general, the correlation was weak with a significant Pearson’s correlation coefficient (*P* < 0.05, uncorrected) only between the attention aggregate score and the ΔCS*_σ_* of VIS(153) (*R* = 0.60, *P* = 0.003), of DAN(77) (*R* = 0.43, *P* = 0.04) and of DMN(33) (*R* = 0.47, *P* = 0.03).

The multiple regression analysis (Fig. 6) also yielded poor description of the dependent variable. For all NPS domains, the relation between prediction and dependent variable was low with *R*^2^ < 0.25 except for the attention aggregate score for which the *R*^2^ was 0.64 (*P* < 10^−11^). A similar result was reached for the precision of the estimates: the obtained CVs were, on average, equal to 237.8% (range: 55.3–774.7%) for language prediction model, 69.2% (range: 21.7–192.1) for the attention model, 239.8% (range: 33.3–578.6%) for the memory model and 441.6% (range: 104.2–1499.5%) for the executive model. According to these findings, the only reliable model for NPS aggregate score prediction was the one developed for predicting the attention performances.

Thus, a statistically significant relationship between the changes in networks topography and NPS performances is supported only for the attention domain. Interestingly, the components whose alterations contribute to this model belonging to networks such as VIS, DAN and DMN that are strongly associated with attention functions.^53^

Demographical variable such as age and education can influence the NPS aggregate score and potentially affect the relationship with RSN alterations. Hence, we performed a multivariate analysis with non-negative sparsity constraints that also included age and education as possible predictors (independent variables). Supplementary Fig. 5 confirms that the attention domain remains the only explained by RSN changes and that age and education do not play a significant role.

## Discussion

The pre-surgical acquisition of task and rs-fMRI is a viable and informative tool to support surgical planning and investigate the effects of brain tumour on human brain. Most studies to-date have focused on the functional mapping of eloquent areas such as sensory-motor areas^14,15^ and LANG network areas^16,54^ or limited the investigation to the perilesional region.^2,7^ Nevertheless, preliminary findings suggested that brain tumour affect broader systems such as brain networks^9,18^ and proposed the lesion-network^55,56^ symptom mapping as an approach to get relevant insight on the impact of these expansive lesions.

Here, we propose a novel method to quantify changes in topography and strength of the RSNs across the whole brain after a high-resolution spatial decomposition based on a HC template. A whole-brain approach of rs-fMRI data allows us to better exploit the richness of the data and yields a detailed description of functional network alterations at the level of individual patients both in the perilesional and in distal regions. This is highly valuable for clinical evaluation. The method was applied to a cohort of tumour patients with heterogeneous tumour location and histopathology.

The most important result is the discovery that a large fraction of the functional connections of the brain are altered in brain tumours. No matter how we aggregate the results, the effect is important. Out of 45 RSN components identified in HCs a mean of 7 ± 5 components were altered. When we aggregate by percentage of patients with affected RSNs, the range across RSNs varies with 71% of the patients showing alteration of the FPN to 8% with alterations of the LANG network. When we average across patients and look at % of altered components in each RSN the range varies from 36% of the components in the FPN to 9% in the LANG network.

Notably, we see no alterations in the SMN and AUD network and very infrequent alterations in the LANG network. The relative predominance of networks with a frontal component (FPN, CON, CCN) is explained by the higher frequency of frontal tumours. The paucity of altered networks in eloquent regions (SMN, AUD, LANG) is due to a sample bias. All our patients underwent surgery and were selected not to suffer postoperative neurological deficits of these functions. In fact, Fig. 2 shows no tumour with a localization in the motor cortex and very few with a localization near Broca area.

However, it is important to highlight, this is the second result, that functional network abnormalities did not fall only in the region of structural damage or even within the much larger oedema region. This can be appreciated in Fig. 3 where individual RSN components near the lesion are partially preserved, whereas those at a distance are affected. It is also evident in Supplementary Table 2 with a mean overlap between RSN alterations and tumour core/necrosis of 2.6 ± 2.3% (range: 0–8.2%) and with oedema of 1.0 ± 1.5% (range: 0–4.9%). About 96.3% of RSN alterations fall within normally appearing structural tissue.

This finding has two very important clinical implications. First, the consistent sparing of RSN component in the cortex overlying the tumour has implications for the surgical approach. Although a straight perpendicular approach may be the shortest path to the subcortical white matter lesion, this approach will hit functional synchronized cortex. More, parallel navigation to subcortical lesions has been proposed using tractography (e.g. by Essayed *et al.*^57^), but our SS fMRI mapping provides a relatively easier way that uses a less operator dependent method.

Second, patients with brain tumours most often present to the clinician because of new onset neurological deficits. A scan typically shows the primary lesion and perilesional white matter oedema. Steroid therapy is started to decrease the extracellular oedema caused by the tumour and patients’ symptoms typically improve. In addition, anticonvulsants for control of epilepsy are also administered. Neurological symptoms are thought to improve due to the local resolution of the oedema. However, our results clearly show that another explanation for the neurological symptoms is the remote dysfunction of functional brain networks in structurally normal areas. Our patients at the time of scan had received already several days of high-dose steroid therapy, and we can only speculate if the effects on the RSN and relative overlap with oedema were enhanced or minimized by the therapy. The likely mechanism of remote functional dysfunction is the disconnection, either structural or functional, caused by the lesion on white matter pathways connecting different nodes of a network. This is evident in Fig. 3, where the affected DMN shows a normal component in the IPL cortex overlying the lesion but no synchronization was observed in anatomically connected regions of the praecuneus, contralateral IPL and frontal cortex. Since gliomas invade along preferential routes, such as those along white matter tracts and in the perineuronal and perivascular spaces,^58^ we can speculate that the lack of distant synchronization reflects a combination of structural and functional effects on white matter pathways. For example, it is now increasingly recognized that neuronal activity robustly regulates central nervous system glial precursor proliferation as part of a process known as myelin plasticity and that gliomas interact with neurons potentially altering neuronal activity.^59^ Our results are in line with previous studies on the long-distance effect of brain tumours on functional connectivity. De Baene *et al*.^60^ using graph theory highlighted that the functional connectivity of the contralesional hemisphere is affected by tumours and that these changes are related to performance of attention and cognitive flexibility tasks. Nenning *et al*.^20^ have reported that unilateral glioblastomas alter inter-hemispherical functional connectivity and that these alterations relate to the proximity of the RSN to the tumour, not strictly its anatomical distance. More recently, Stoecklein *et al*.^21^ developed a global functional index based on voxel-wise functional connectivity to quantify altered connectivity locally at the individual level. They showed abnormalities in structurally normal tissue both in the lesioned and normal hemisphere, especially in high-grade tumours.

We found that RSN alterations showed some relationship with neuropsychological deficits, especially visuospatial attention in line with Nenning *et al*.^20^ and that DMN alterations were strongly linked to cognitive dysfunction, as previously reported by Kocher *et al*.^61^ and Stoecklein *et al*.^21^ We think that the low prediction power in this study for other functional domains can be imputed to the low number of subjects. Previous studies in patients with brain tumour have reported functional network characteristics associated with cognitive functioning (see the studies by Ghinda *et al*.,^2^ Fox *et al*.^9^ and Hacker *et al*.^18^ for a review), but the analyses were restricted to specific RSNs or overall network graph properties^17,20,60^ making the statistics simpler.

Despite promising results, this study has some limitations. The relatively small number of subjects prevents sensitive behavioural correlations. The genetic profile of the tumours that certainly have an impact on prognosis and likely brain organization was not considered given the small sample size. The heterogeneity of the topography of the lesions that could not be quantitatively compared with the RSN altered topography. The uncomplete full coverage of the resting-state acquisition prevented us to study also these connections that have been mainly related in recent studies to language dysfunctions in glioma patients.^54,62,63^

In summary, in this study, we developed a novel method to investigate the changes in RSNs in glioma patients. We showed that functional alterations in network topography and strength are widespread and occur far from the lesion or the oedema, with cortical regions near the glioma that are potentially preserved. Our individualized approach could identify cortical regions to be carefully navigated during surgery, and widespread alterations of functional networks are away from the tumour that contribute to cognitive disability.

## Supplementary Material

fcac082_Supplementary_Data

## Abbreviations

AUD =: auditory network
CCN =: cognitive control network
CON =: cingulo-opercular network
CS =: cosine similarity
CV =: coefficient of variation
DAN =: dorsal attention network
DMN =: default mode network
EPI =: echo planar imaging
FA =: flip angle
fMRI =: functional MRI
FOV =: field of view
FPN =: fronto-parietal network
FRN =: frontal network
GIFT =: Group ICA of fMRI toolbox
HCs =: healthy controls
IC =: independent component
ICA =: independent component analysis
IPL =: inferior parietal lobule
LANG =: language network
MBAccFactor =: multiband acceleration factor
NPS =: neuropsychological score
rs-fMRI =: resting-state functional MRI
RSN =: resting-state network
SMN =: sensorimotor network
T1w =: T_1_-weighted image
TE =: echo time
TM =: tumour mask
TM + O =: tumour and oedema area mask
TR =: repetition time
VIS =: visual network.
