# Supplementary material for: Widespread cortical functional disconnection in gliomas: an individual network mapping approach
Source: Brain Commun. 2022 Apr 8;4(2):fcac082. doi: 10.1093/braincomms/fcac082 (PMC9034119; doi:10.1093/braincomms/fcac082)

Supplementary Materials

**Widespread cortical functional disconnection in brain gliomas: an individual network mapping approach**

Silvestri E., Moretto M., Facchini S., Castellaro M., Anglani M., Monai E., D’Avella D., Della Puppa A., Cecchin D., Bertoldo A., and Corbetta M.

**Supplementary Table 1:** Single patient’s demographical and clinical information


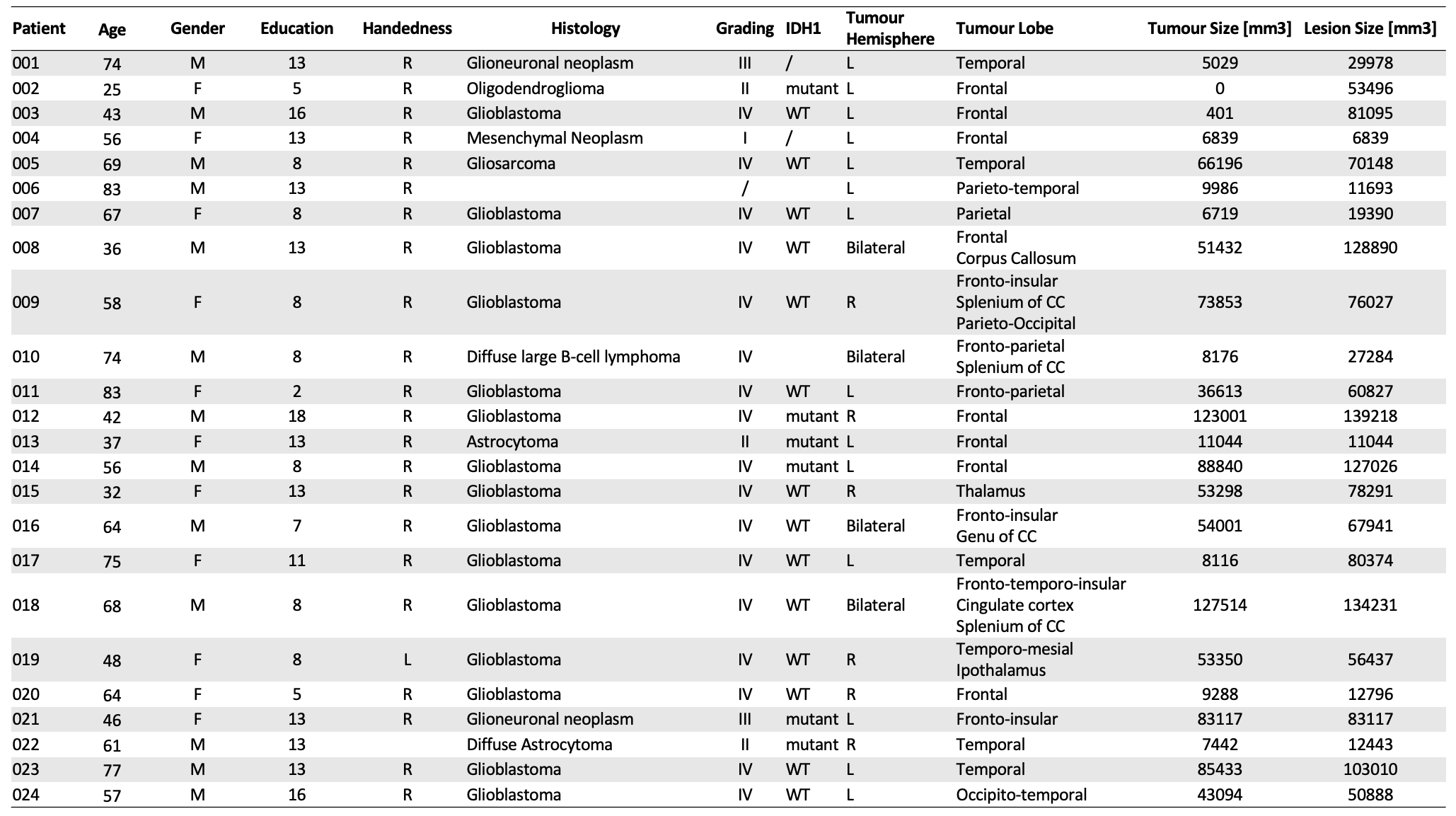


**Supplementary Figure 1:** Outline of the pipeline followed for each patient to build the “expected” abnormal RSNs mask and calculate the spatial overlap with tumour, oedema, and normal appearing areas. In a representative patient, four components resulted to be altered. For each altered component we extracted from the template the expected pattern of the component (over imposed in red to the patient’s FLAIR structural image, second box) and create a mask of expected abnormal RSNs as the union of the four masks. Then the overlaps between the abnormal RSNs mask and oedema, tumour core and normal appearing tissues were computed exploiting the individual manual segmentations (Tumour Mask in the figure).

**
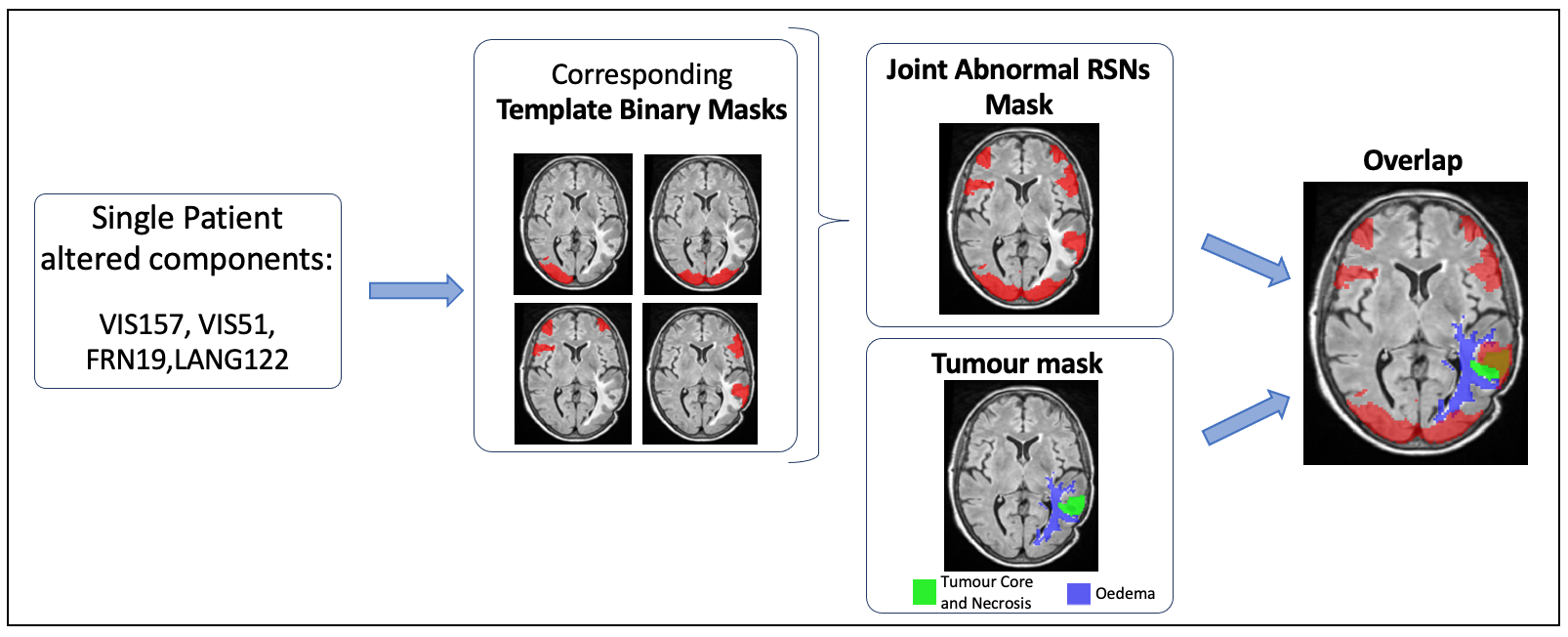
**

**Supplementary Figure 2:** Spatial pattern of the peak activation clusters of the group independent components superimposed on the T1w image of the MNI152 symmetric atlas (in grey scale). Peak activation clusters are calculated by keeping only voxels with a z-score value greater than 5 and belonging to connected clusters of at least 200 voxels. Images are displayed in radiological convention.


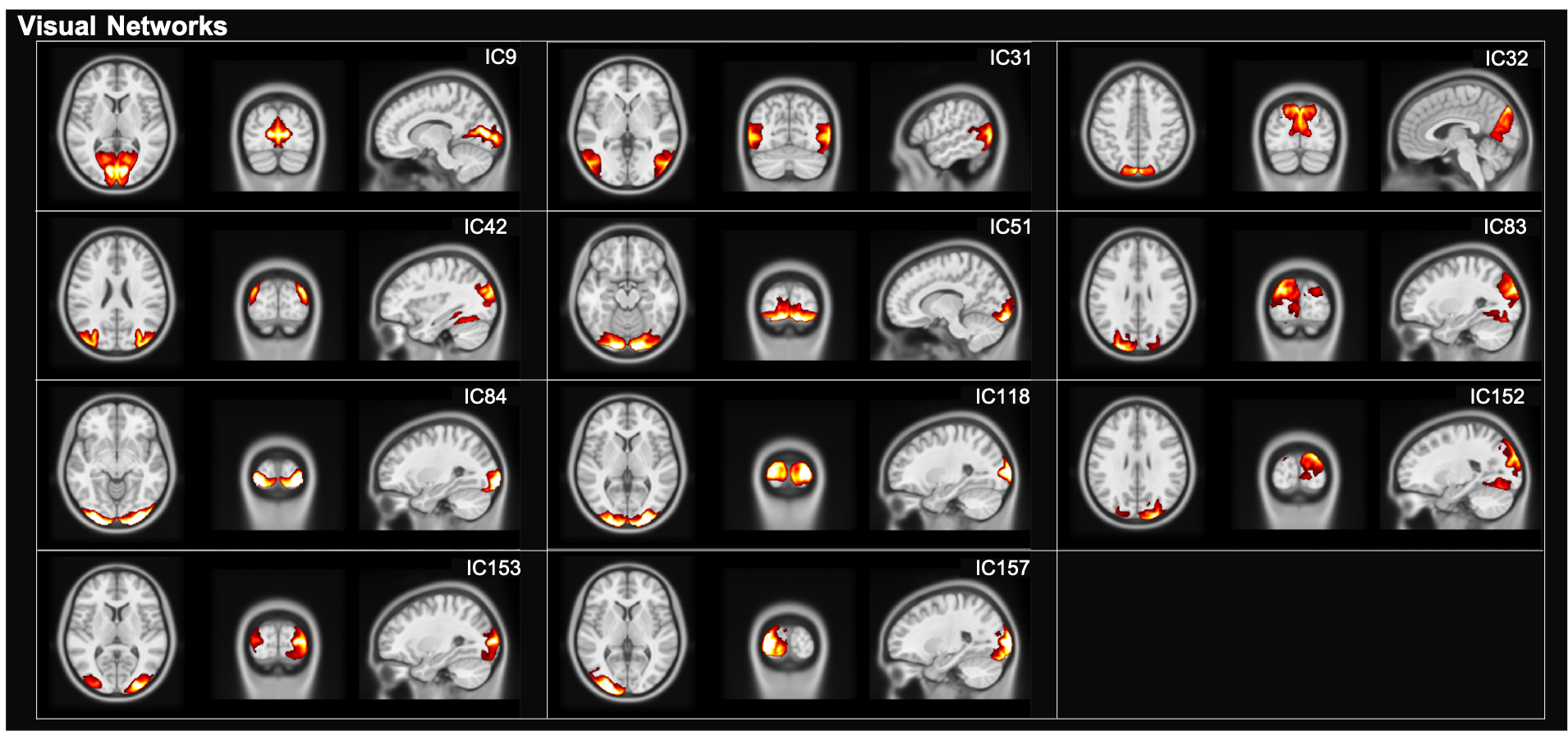


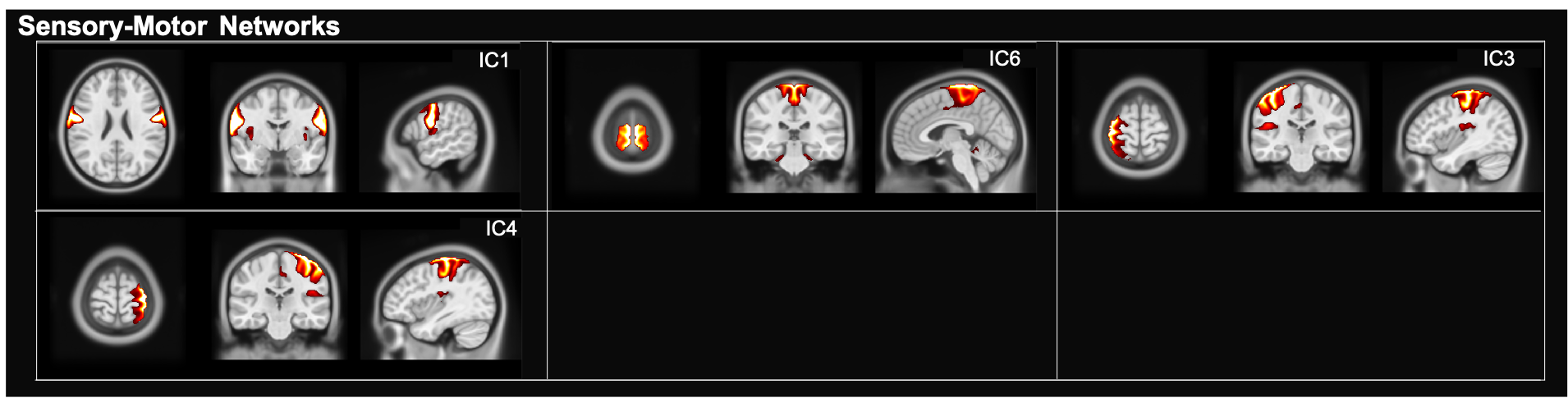


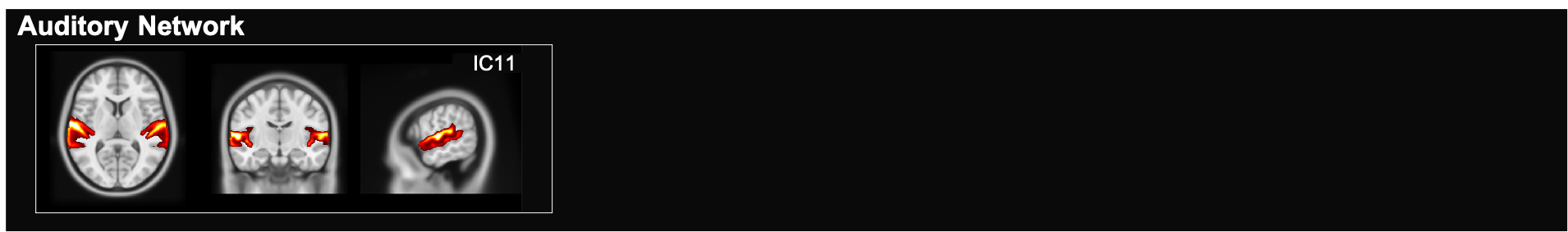


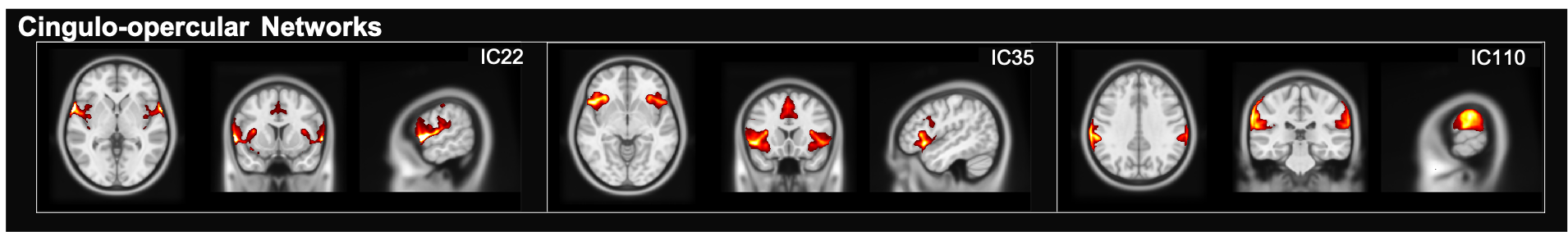


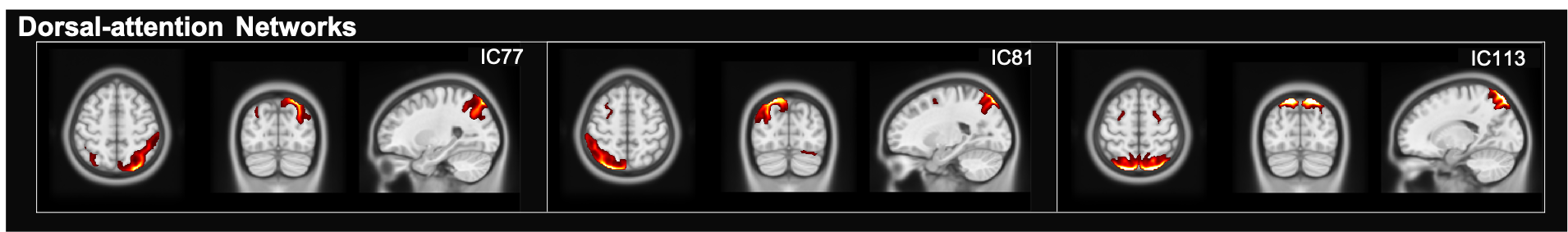


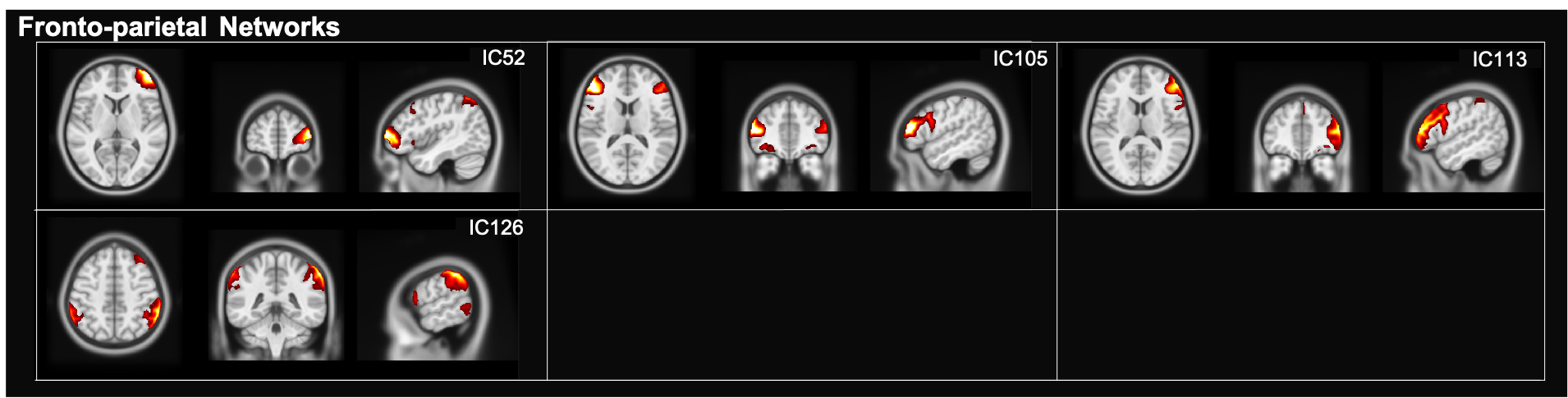


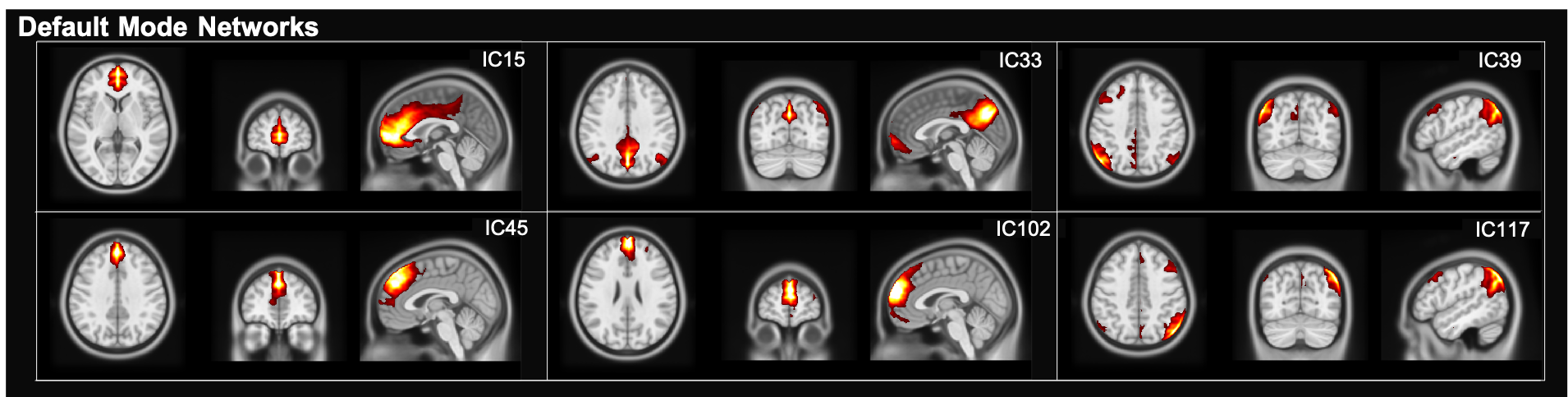


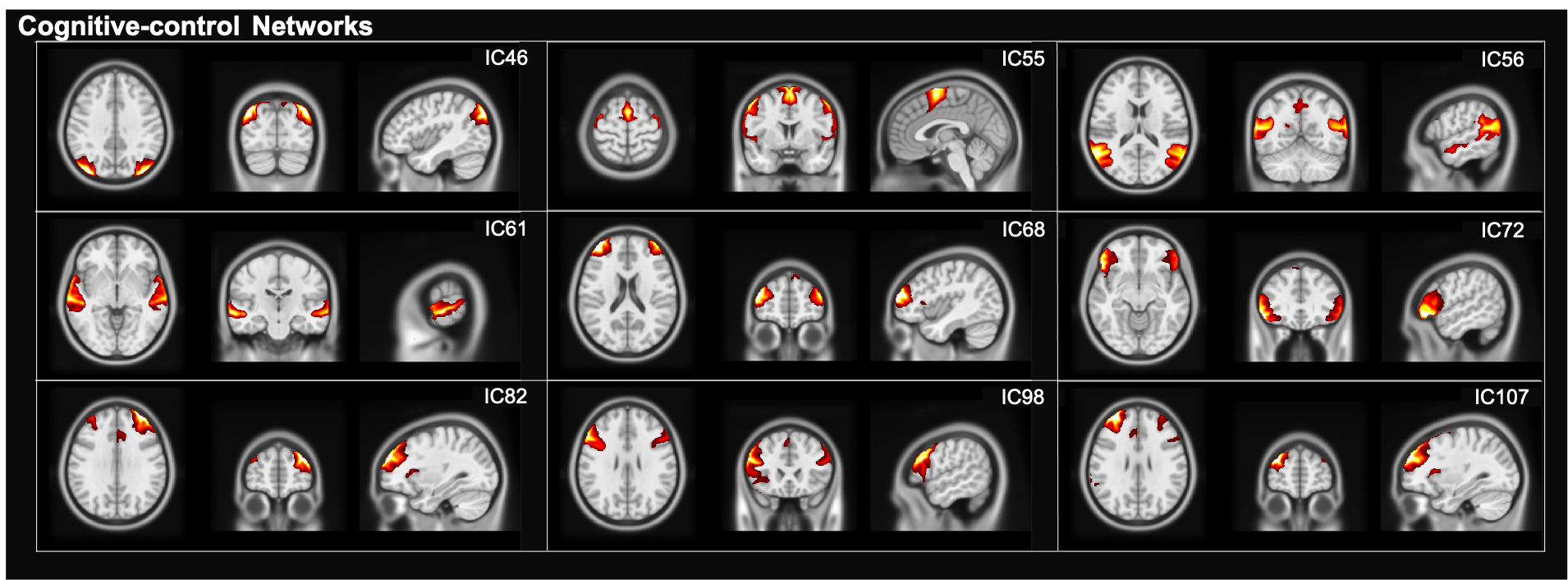


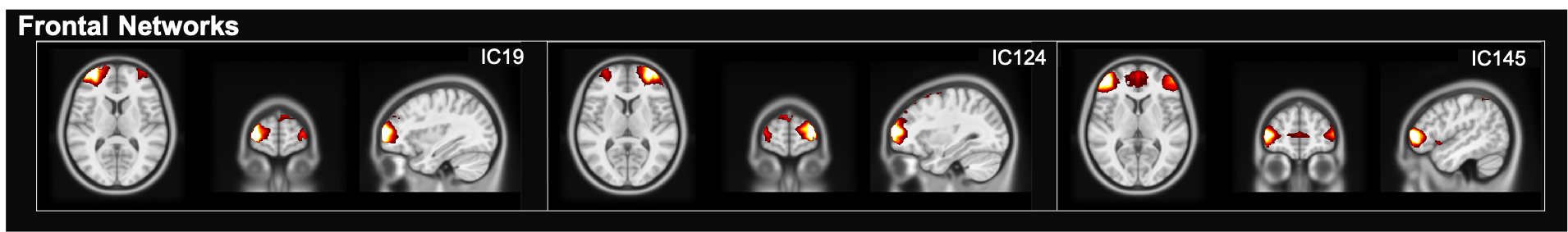


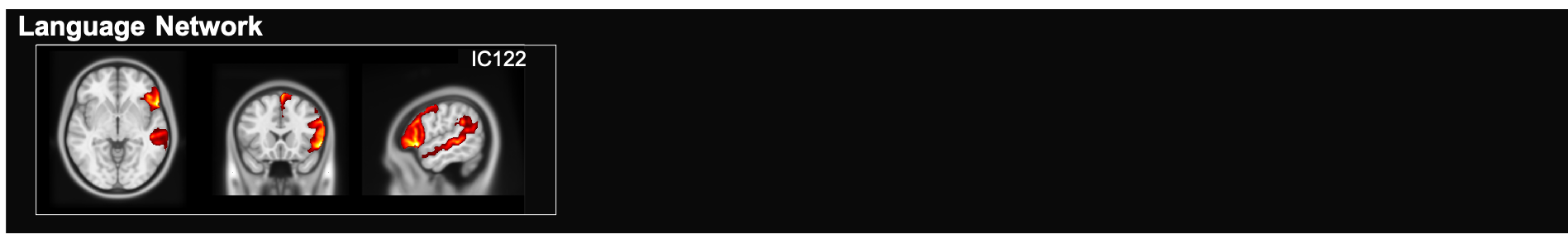


**Supplementary Figure 3:** Percentage of spatial overlap between altered components and three different tissues: tumour mask (A), oedema (B) and normal appearing brain tissues (C).


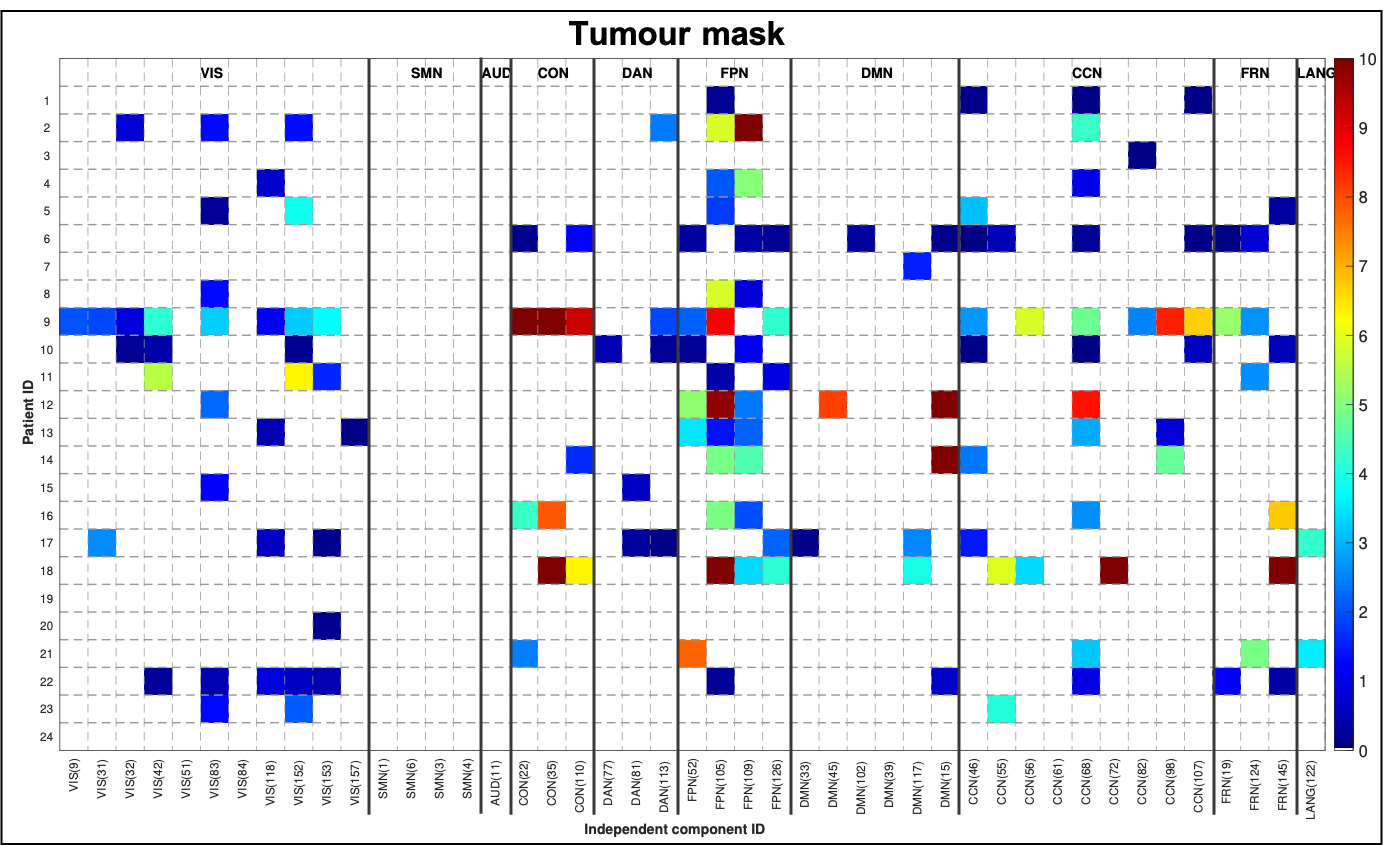


Panel A


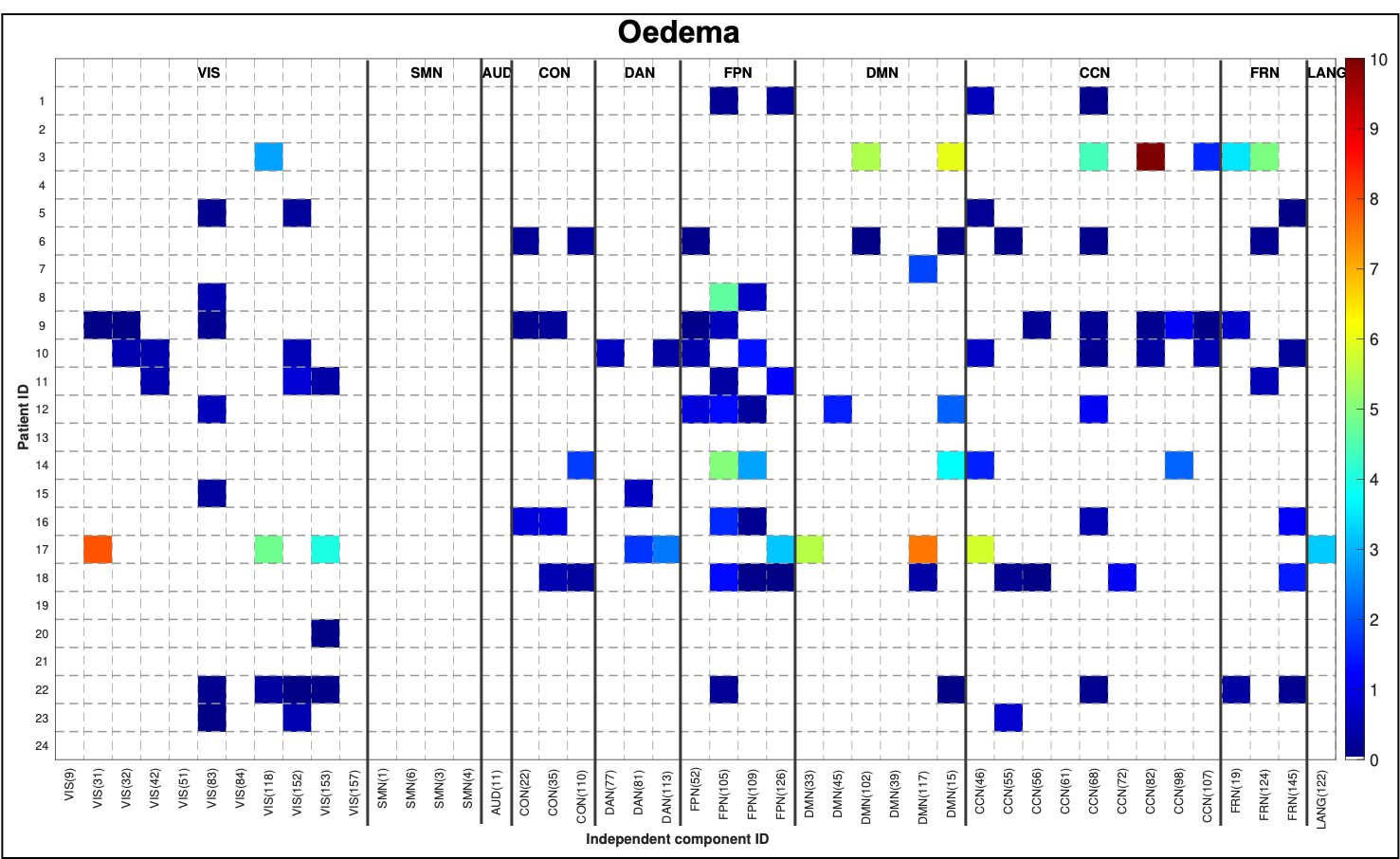


Panel B


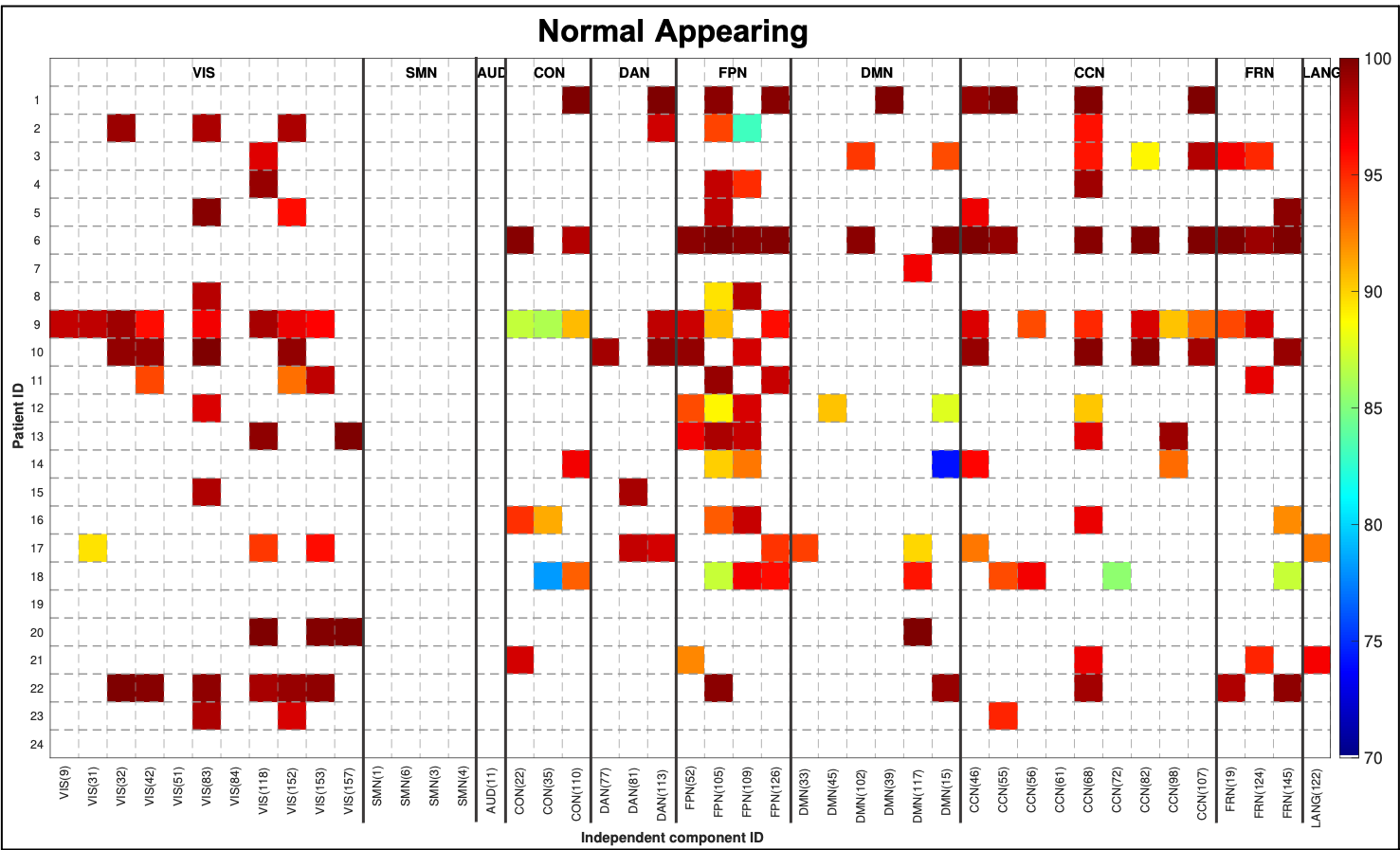


Panel C

**Supplementary Table2:** Single patient percentage overlap between the altered components and 1) the tumour mask (core and necrosis), 2) the oedema and 3) the remaining normal-appearing brain tissues


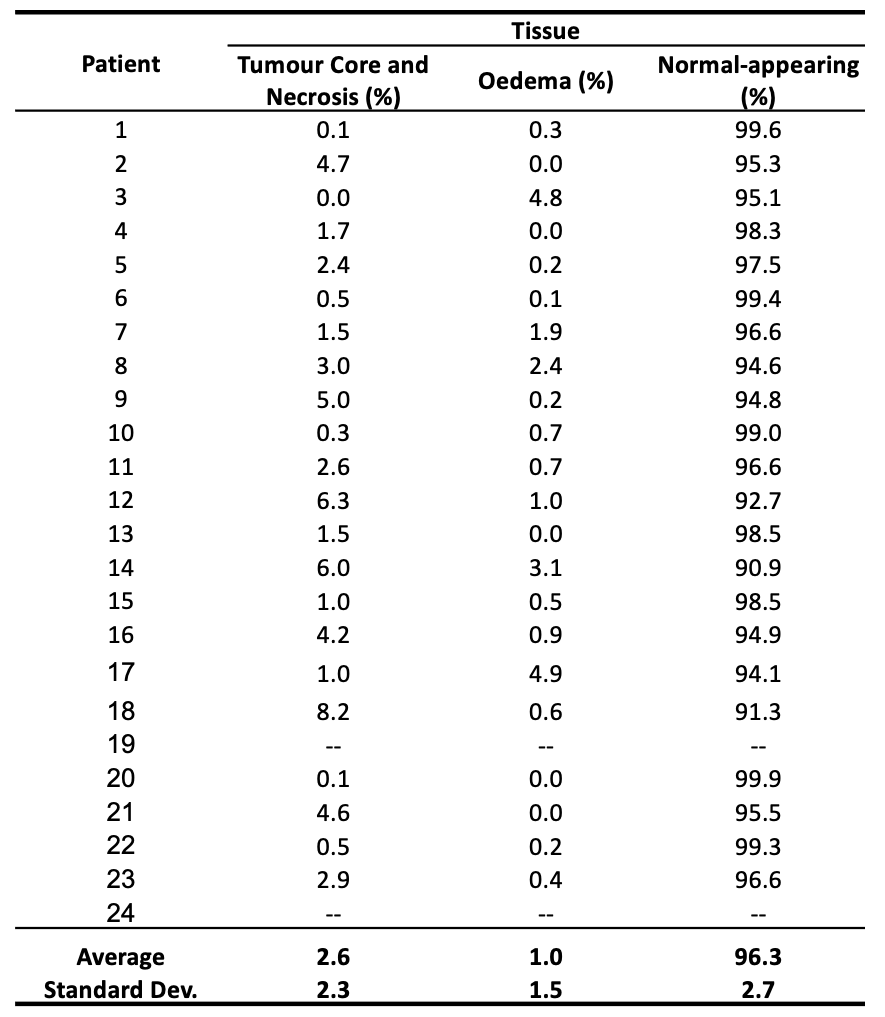


**Supplementary Table 3:** Neuropsychological tests grouped in the four considered functional domains. OCS= Oxford Cognitive Screen.


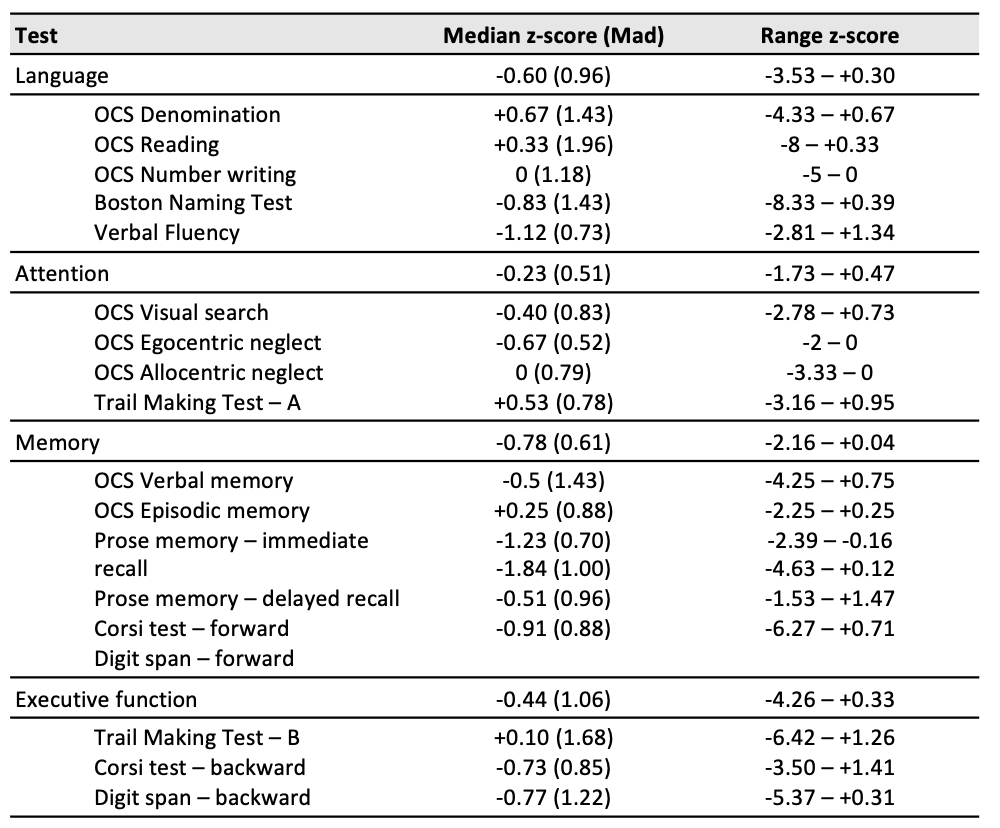


**Supplementary Figure 4:** Distribution of aggregate neuropsychological scores in the patient population. Med=median, MAD=mean absolute deviation, SD=standard deviation.


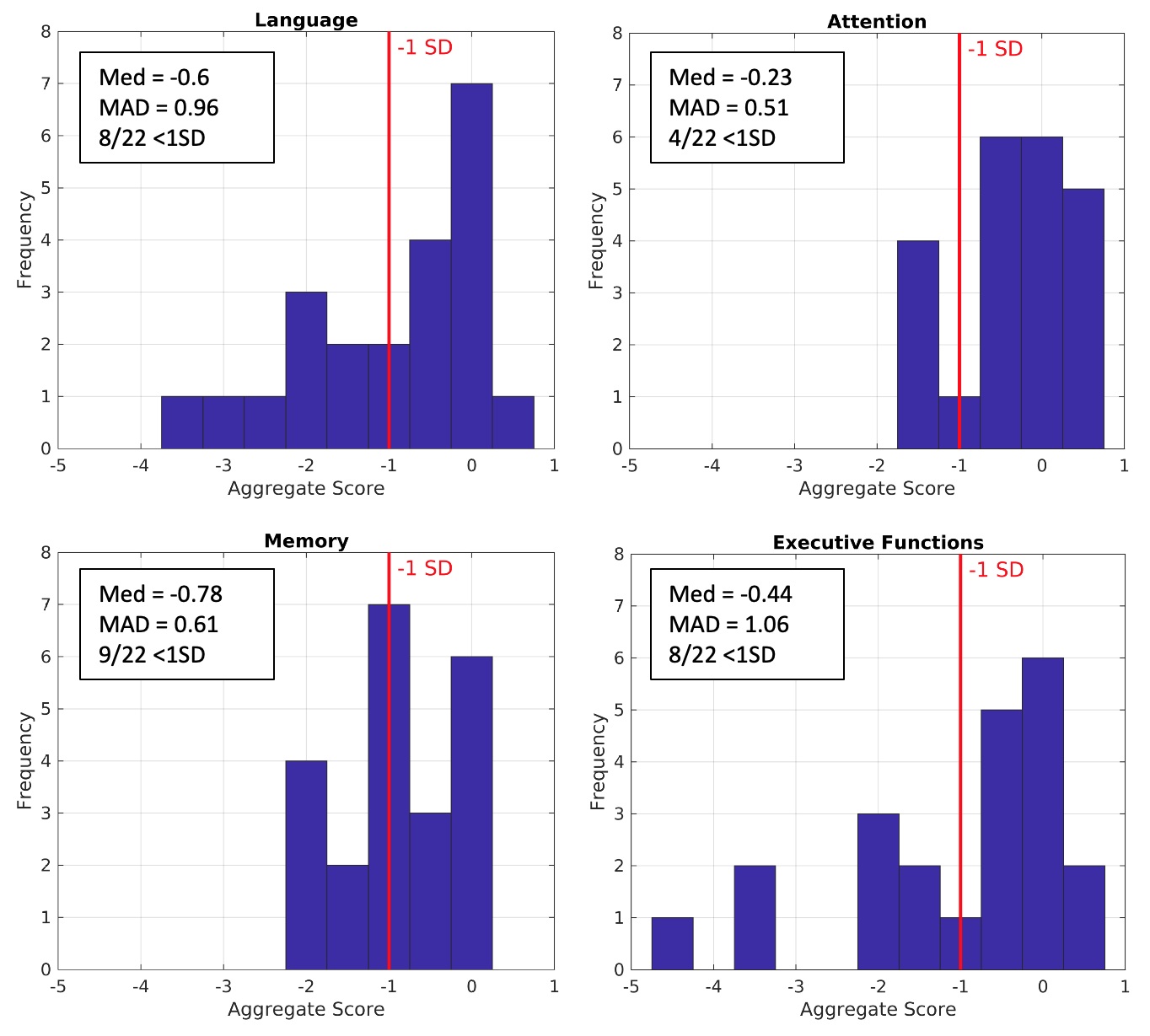


**Supplementary Figure 5:** Relationship between changes in component’s spatial pattern and neuropsychological aggregate scores. On the left, the matrix reports the correlation between component’s delta cosine similarity -ΔCSσ (on the rows, with group spatial pattern of the component on the left), age and education, and neuropsychological (NPS) aggregate scores (on the columns) for the components and demographical information selected with the multivariate analysis for at least one functional domain. In grey scale, only the correlation values obtained for predictors included in the linear model are reported. On the right, the four boxes show the observed versus predicted aggregate scores (black dots) and their expected linear relationship (i.e., unitary slope, grey line) for the four tested neuropsychological functional domains.


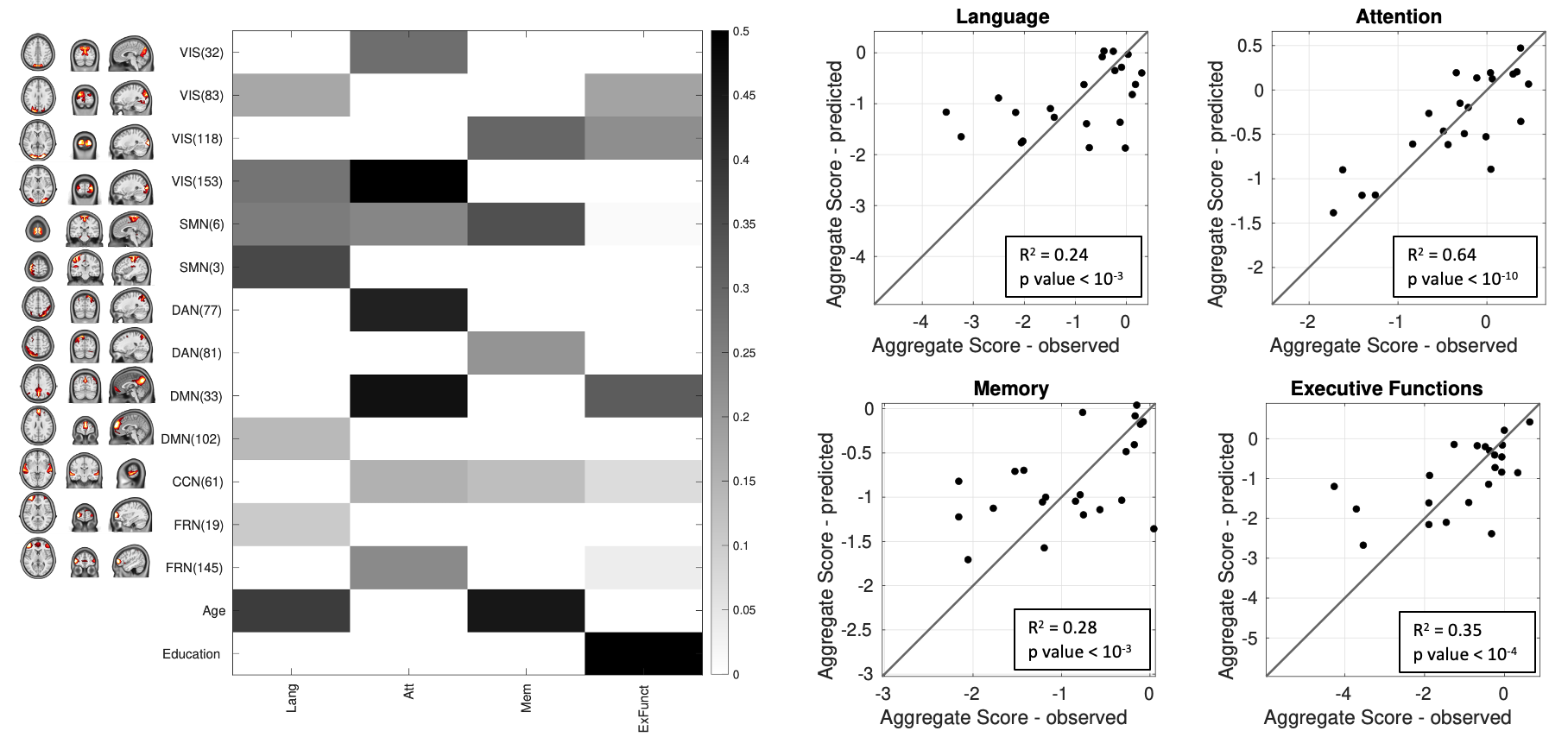

Supplement: fcac082_Supplementary_Data [file fcac082_supplementary_data.docx]
